# Targeting Microtubule-Associated Protein Tau in Chemotherapy-Resistant Models of High-Grade Serous Ovarian Carcinoma

**DOI:** 10.3390/cancers14184535

**Published:** 2022-09-19

**Authors:** Maria V. Barbolina

**Affiliations:** Department of Pharmaceutical Sciences, College of Pharmacy, The University of Illinois at Chicago, 833 South Wood Street, Chicago, IL 60091, USA; mvb@uic.edu

**Keywords:** ovarian cancer, chemoresistance, paclitaxel resistance, tau, leucomethylene blue, TRx0237

## Abstract

**Simple Summary:**

Resistance to chemotherapy in patients with high-grade serous ovarian carcinoma is a clinically relevant problem, and it results in high mortality. The aim of the study was to determine whether targeting microtubule-associated protein tau could reduce cell proliferation and tumor burden in cell culture and mouse models of chemotherapy-resistant high-grade serous ovarian carcinoma. To achieve this aim, I tested an inhibitor of tau, leucomethylene blue, which was originally developed against neurodegenerative diseases, and found that it efficiently reduced cell and tumor growth. Additionally, paclitaxel and leucomethylene blue synergized in reducing cell growth and tumor burden. Altogether, the data demonstrate that tau could be considered a new target for treatment of chemotherapy-resistant high-grade serous ovarian carcinoma, and leucomethylene blue could potentially add to the arsenal of drugs to treat the terminal stage of this deadly malignancy.

**Abstract:**

Relapsed, recurrent, chemotherapy-resistant high-grade serous ovarian carcinoma is the deadliest stage of this disease. Expression of microtubule-associated protein tau (tau) has been linked to resistance to paclitaxel treatment. Here, I used models of platinum-resistant and created models of platinum/paclitaxel-resistant high-grade serous ovarian carcinoma to examine the impact of reducing tau expression on cell survival and tumor burden in cell culture and xenograft and syngeneic models of the disease. Tau was overexpressed in platinum/paclitaxel-resistant models; expression of phosphoSer396 and phosphoThr181 species was also found. A treatment with leucomethylene blue reduced the levels of tau in treated cells, was cytotoxic in cell cultures, and efficiently reduced the tumor burden in xenograft models. Furthermore, a combination of leucomethylene blue and paclitaxel synergized in eliminating cancer cells in cell culture and xenograft models. These findings underscore the feasibility of targeting tau as a treatment option in terminal-stage high-grade serous ovarian cancer.

## 1. Introduction

Epithelial ovarian carcinoma (EOC) remains the deadliest gynecologic malignancy, claiming over 14,000 lives each year in the US and over 207,000 lives worldwide [1,2], mainly due to its peritoneal metastasis. EOC is a complex disease, originating from multiple precursor cells within the reproductive tract [3,4,5]. Among its subtypes, high-grade serous carcinoma of tubo-ovarian origin (HGSOC) is the most common and deadliest [6].

The standard of care for metastatic HGSOC includes surgery and chemotherapy consisting of a platinum drug and a taxane. The molecular mechanisms of these two types of chemotherapy are fundamentally different but complementary in inducing cell death in proliferating cells. Platinum chemotherapy targets DNA during the S-phase, and taxanes disrupt mitotic spindle formation during the M-phase of the cell cycle. Most cases are initially very sensitive to this chemotherapy; however, the treatment often fails due to quickly developing chemoresistance. Options in the second through fourth lines of chemotherapy include other DNA damage and microtubule-targeting agents, including weekly paclitaxel, doxorubicin, topotecan, and inhibitors of poly(ADP-ribose) polymerase, which demonstrate limited efficacy [7,8,9,10]. Unfortunately, all these options demonstrate limited efficacy in a small subset of patients and, eventually, also fail [7,8,9,10]. Chemoresistance is a major obstacle for the success of chemotherapy in the clinic. Previous studies have focused on discovery of the mechanisms of resistance to platinum and taxane chemotherapy and have resulted in identification of the role of alterations in drug accumulation inside the cell, enhancement of DNA repair mechanisms and cell survival pathways, and changes in the tumor microenvironment in chemoresistant state [11,12,13,14,15,16,17,18,19]. However, so far, these studies have not translated into the clinic, and the relapsed, recurrent, chemotherapy-resistant disease is the terminal stage of HGSOC, indicating the dire need for new therapies.

Microtubule dynamics are essential during the cell cycle as separation of sister chromatids depends on effective and timely assembly and disassembly of the mitotic spindle for which alpha and beta subunits of tubulin are the main building blocks. Importantly, microtubule-associated proteins are essential for formation, stabilization, and bundling of microtubules [20]. One such protein, microtubule-associated protein tau (MAPT, tau), is thought to play many roles in formation and functioning of microtubules. It was originally discovered as a protein essential in the process of microtubule polymerization, and it was reported that tubulin did not assemble into microtubules in the absence of tau in vitro [21,22]. Since its initial discovery in the brain, expression of tau has been reported in peripheral tissues as well [23,24]. Tau is important for bundling of microtubules and connecting microtubules with actin cytoskeleton [25]. Depletion of tau in vivo resulted in reduction in the microtubule mass, and it was found that tau associated mainly with the labile plus ends of the microtubules and regulated their length [26]. Depletion of tau in a model of glioblastoma resulted in a significant increase in survival [27]. In epithelial ovarian cancer, it has been reported that low or no tau expression significantly correlated with better progression-free and overall survival [28], suggesting that reduction in tau could be beneficial for increasing life expectancy in patients.

More recently, tau has emerged as a potential biomarker of response to taxanes in cancer treatment [28,29,30,31,32,33,34,35,36,37,38,39,40]. The original report linking expression of tau to paclitaxel response in cancer treatment was published by a breast cancer research group; using an unbiased high-throughput approach, the authors identified tau as the most differentially expressed gene associated with a complete response to preoperative paclitaxel-containing chemotherapy [29]. Since then, expression of tau was found to correlate with a response to paclitaxel in many studies in breast, ovarian, stomach, bladder, and lung cancers [28,29,30,31,32,33,34,35,36,37,38,39,40]. In fact, in epithelial ovarian cancer, it has been reported that low or no tau expression significantly correlated with a response to paclitaxel/platinum chemotherapy [28]. We and others have demonstrated that ectopic modulation of tau expression in cancer cell culture models correlated with a response to taxanes [37,41]. Mechanistically, low expression of tau is thought to correlate with a better response to paclitaxel because they bind to the same binding site on the inner side of the microtubule [29,42,43]. Altogether, previous studies suggest that a reduction in tau in ovarian cancer could result in abrogation of the cell cycle and sensitization to paclitaxel.

Fortunately, tau has been extensively studied in its role in tauopathies, a heterogeneous group of neurodegenerative diseases currently affecting nearly 30 million people [44,45]. The hallmark of tauopathies is the abnormal metabolism of misfolded tau protein, which accumulates outside the cell and forms neurofibrillary tangles. Hyperphosphorylated tau loses its ability to attach to microtubules and gains the capacity to be secreted in the extracellular space and self-aggregate, leading to formation of neurofibrillary tangles [46]. Phosphorylation of tau on S396 results in a reduction in tau affinity to microtubules and was suggested to be a key step in the development of neurofibrillary pathology in brains with Alzheimer’s disease [47,48]. Elevated levels of phosphorylated Thr181 and Tyr231 in cerebrospinal fluid and plasma of patients with neurodegenerative diseases are used as biomarkers of disease progression [49,50]. It has been suggested that phosphorylation of Tyr231 is critical for tau assembly into paired helical filaments, while phosphorylation of Ser396 and Thr181 is also required for tau self-assembly [51]. Interestingly, tau hyperphosphorylation has also been reported in prostate cancer, although its physiological role remains to be determined [52]. For its putative role in neurodegeneration, tau has been extensively investigated as a target in these diseases. Several drugs and other therapeutics have emerged from these efforts [53,54,55], and one such compound, a phenothiazine leucomethylene blue (or 3-N,3-N,7-N,7-N-tetramethyl-10H-phenothiazine-3,7-diamine; developed as TRx0237), is a tau-degrading drug [56,57]. TRx0237 at 8–250 mg/day for up to 34 months has been evaluated for safety and efficacy in multiple phase II and III clinical trials in patients with Alzheimer’s disease and frontotemporal dementia (NCT01626391, NCT01689233, NCT03446001, NCT01626378, NCT01689246, NCT02245568) and found to have an acceptable side effect profile, with about 80% of patients displaying non-life-threatening side effects, and less than 2% all-cause mortality. Previous studies demonstrated that phenothiazines, including TRx0237 and methylene blue, result in proteolytic degradation of tau via both lysosomal and proteasomal degradation [56,57,58,59,60]. TRx0237 has undergone phase III clinical trials in patients with advanced Alzheimer’s disease and frontotemporal dementia and is currently undergoing a phase III clinical trial in patients with early Alzheimer’s disease. The drug was demonstrated to be fairly safe, increasing the interest in testing its efficacy in models of HGSOC. I used TRx0237 to examine whether targeting tau in models of chemotherapy-resistant HGSOC could result in reducing tumor burden and sensitization to paclitaxel.

## 2. Materials and Methods

### 2.1. Cell Lines

Human-derived epithelial ovarian carcinoma cell line OVCAR4 was obtained from the NCI Tumor Cell Repository (Detrick, MD, USA). FVB/N mouse-derived epithelial ovarian cancer cell line STOSE was obtained from Dr. Vanderhyden (The Ottawa Hospital Research Institute, Ottawa, ON, Canada). All cell lines were cultured as suggested by manufacturers for no longer than 15 consecutive passages and routinely assessed by cell morphology and average doubling time; identity of the OVCAR-4 clones was confirmed using a short tandem repeat analysis that indicated 100% match of cell-line-specific DNA loci to the tested samples. The cell lines were free from contamination by *Mycoplasma fermentans*, as determined using The LookOut Mycoplasma PCR Detection kit (Sigma, St. Louis, MI, USA).

### 2.2. Mice

Female 6–8-weeks-old athymic nude—FOXN1^NU^ and FVB/N mice were obtained from Envigo (Indianapolis, IN, USA). All experimental procedures were performed accgooording to the Institutional Animal Care and Use Committee protocol approved by the Animal Care Committee of UIC.

### 2.3. Antibodies and Reagents

Rabbit anti-human tau ab-3 was obtained from ThermoScientific (Fremont, CA, USA). Mouse anti-tau antibody (5C6) was purchased from Iowa Developmental Studies Hybridoma Bank (Iowa City, Iowa, IA, USA). Rabbit monoclonal anti-human vinculin, mouse anti-human β1-tubulin antibodies, goat anti-mouse IgG AlexaFluor Plus488, ProLong Gold antifade reagent with DAPI, and AlexaFluor546 phalloidin were obtained from Invitrogen (Waltham, MA, USA). Goat anti-rabbit IgG DyLight488 antibodies were purchased from Vector Laboratories (Newark, CA, USA). Mouse anti-human EGFR, mouse anti-human GAPDH, scrambled siRNA, and a pool of tau-specific siRNAs were obtained from Santa Cruz Biotechnology (Dallas, TX, USA). Rabbit anti-human tau phosphoSer396, rabbit anti-human phosphoTyr1086 EGFR, and mouse anti-human ALDH1A1 were obtained from R&D Systems (Minneapolis, MD, USA). Mouse anti-human phosphoThr181 tau and mouse anti-human phosphoThr231 tau antibodies were obtained from BioLegend (San Diego, CA, USA). Anti-mouse and anti-rabbit Azure490, Azure 550, and Azure650 secondary antibodies were obtained from Azure Biosystems (Dublin, CA, USA). Mouse brain extract and mouse anti-human CD44 antibody were from Novus (Centennial, CO, USA). Normal human brain and Alzheimer’s disease human brain extracts were obtained from GeneTex (Irvine, CA, USA). Molecular weight marker for Western blot was obtained from ApexBio (Houston, TX, USA). Tau ladder, WST-1 cell proliferation assay kit, DuoLink Proximity Ligation assay kit, and paclitaxel were obtained from Sigma-Aldrich (St. Louis, MO, USA). TRx0237 was obtained from MedChemExpress (Monmouth Junction, NJ, USA). Tau ELISA was obtained from Biomatik (Wilmington, DE, USA). Specimens of ascites from HGSOC were purchased from Proteogenix (Inglewood, CA, USA) (Table S2). Chromogenic Western Blot Dual kit was purchased from Enzo (Farmingdale, NY, USA).

### 2.4. Immunofluorescence Staining and Imaging

Immunofluorescence staining and imaging with AxioVert Zeiss fluorescence microscope was conducted as detailed in Refs. [25,26,32]. Tau antibodies were used at 1:100–1:250 dilution and β1-tubulin antibodies were used at 1:250 dilution. Mouse AlexaFluor488 and rabbit DyLight488 secondary antibodies were used at 1:250 dilution.

### 2.5. Duolink Proximity Ligation Assay

Duolink proximity ligation assay for detection of interaction between tau and β-tubulin was conducted using rabbit anti-human tau ab-3 antibodies, mouse anti-human β-tubulin antibodies, Duolink^®^ In Situ PLA^®^ Probe Anti-Rabbit PLUS, Duolink^®^ In Situ PLA^®^ Probe Anti-Mouse MINUS, and Duolink^®^ In Situ Detection Reagents Green as suggested by the manufacturer (Sigma-Aldrich). Tau Ab-3 and β1-tubulin antibodies were used at 1:100 dilution.

### 2.6. ELISA

ELISA was conducted using total tau kit (Cat# EKF57422, Biomatik) as suggested by the manufacturer.

### 2.7. Spheroid Formation

Spheroids were generated using an agarose overlay method described previously [61,62,63].

### 2.8. Western Blot

Procedures were conducted as described in Refs. [64,65,66]. The proteins were visualized using either fluorescence-based detection and Azure400 Imager or chromogenic-based detection and EPSON scanner. Each blot was conducted at least four times.

### 2.9. Cell Proliferation

Cell proliferation was performed using WST-1 method as described in Refs. [67,68].

### 2.10. Transwell Cell Migration

Transwell cell migration was conducted as previously described [64,65,69]. Briefly, cells were seeded atop filters with 8-micron pore size and allowed to migrate for 9 h.

### 2.11. Proteome Profiler Human Phospho-Kinase Array

Proteome profiler human phospho-kinase array kit (R&DSystems) was used according to the manufacturer’s suggestions. Untreated OVCAR-4 and OVCAR4-PR2 as well as OVCAR-4 treated with 13.3 µM TRx0237 (equivalent of 2 × IC50) for 72 h were used to conduct gene expression and activation analyses. Membranes were imaged using chemiluminescence imaging and Azure400 imaging system.

### 2.12. Tumor Formation and Treatments

To create intraperitoneal tumor lesions, 10^6^ OVCAR-4 (or clone) or STOSE (or clones) cells were injected intraperitoneally (i.p.) into athymic nude mice or FVB/N mice. The animals were treated, or not, with either paclitaxel or TRx0237 or their combination. Paclitaxel was dissolved in absolute EtOH, Kolliphor EL, and normal saline (0.9% NaCl) to 2 mg/mL, sterilized by passing through 0.22 micron filter, and administered i.p. using 28 gauge needle in a volume of 200 µL twice weekly. TRx0237 was dissolved in water to 2.5–5 mg/mL, as needed, sterilized by passing through 0.22 micron filter, and administered by oral gavage using plastic feeding tubes in a volume of 200 µL either twice weekly or 5 days a week depending on experimental design. STOSE-bearing animals were monitored three times weekly until they reached humane endpoints and sacrificed. OVCAR-4-bearing animals were monitored three times weekly and were sacrificed 3 months after tumor inoculation. Tumors were excised, weighed, fixed in paraformaldehyde, and paraffin-preserved as described before [63,67,69]. Peritoneal carcinomatosis index was found by assessing the number and size of abdominal lesions using a previously published procedure [70].

To create subcutaneous tumors, OVCAR-4 or OVCAR4-PR2 at 100, 1000, or 10,000 cells/mouse were injected into either right or left flanks and observed for tumor formation twice weekly for 15 months.

### 2.13. Statistics

Comparisons between two datasets with normal distributions were conducted using Student’s *t*-test and Microsoft Excel software. Mann–Whitney U test was used to compare two datasets with abnormal distribution. Differences were considered statistically significant at *p* < 0.05.

## 3. Results

### 3.1. Generation of Paclitaxel-Resistant Cell Lines

My goal was to identify an effective treatment against chemotherapy-resistant models of HGSOC, including platinum- and platinum/paclitaxel resistant ones, as they represent the most advanced stages of relapsed, metastatic HGSOC. For this purpose, I selected a human-derived HGSOC cell line (OVCAR-4) and a mouse-derived serous ovarian cancer cell line (STOSE), both of which are tumorigenic in vivo and carboplatin-resistant (Supplementary Table S1). Genomic analysis of OVCAR-4 indicated that this cell line belongs to the HGSOC histotype [71]; the cell line was developed from a patient resistant to platinum chemotherapy, and it carries a clinically relevant mutation in TP53 (Leu130Val) [72,73] and replicates the pattern of metastatic colonization in the peritoneal cavity, similar to the human disease [74,75]. STOSE was derived from mouse ovarian surface epithelium, one of the putative sites of origin of HGSOC [76]. This cell line carries wild type TP53, similar to 6% of all HGSOC cases [77], and replicates metastatic colonization in the peritoneal cavity, similar to the human disease, and accounts for the role of the immune system in tumor progression and treatment response.

To convert these platinum-resistant cell lines into platinum/taxane resistant models, I adapted a previously described methodology to develop clinically relevant paclitaxel-resistant cell culture models of HGSOC [78]. Cell lines OVCAR-4 and STOSE were subjected to a 3-h treatment with paclitaxel at 12 nM (OVCAR-4) and 25 or 50 nM (STOSE) once weekly for six weeks and allowed to recover in drug-free complete media between the treatments. This pulse-selection approach was used to mimic the drug exposure pattern in patients, and the paclitaxel concentration used was the highest that permitted survival and selection of resistant clones. One OVCAR-4-based clone was selected and termed OVCAR4-PR2. Five clones were generated using STOSE treated with 25 and 50 nM paclitaxel, designated STOSE-PR25, STOSE-PR25-3, STOSE-PR50, STOSE-PR50-2, and STOSE-PR50-3, respectively. I determined IC50 of paclitaxel in parental cell lines and their subclones and found that STOSE was originally more resistant to paclitaxel compared to OVCAR-4 (Supplementary Table S1). Importantly, selected subclones of OVCAR-4 and STOSE were characterized by 2.5–5-fold increase in IC50 for paclitaxel in comparison to their corresponding parental cell lines (Supplementary Table S1). I selected OVCAR4-PR2, STOSE-PR25, and STOSE-PR50 to test whether these subclones were more paclitaxel-resistant in vivo in comparison to the parental cell lines. Cells were i.p. injected into mice abdomens and allowed to lodge peritoneal lesions for 1 month (OVCAR-4 and OVCAR4-PR2) or 2 weeks (STOSE, STOSE-PR25, and STOSE-PR50), followed by a treatment with twice weekly i.p. paclitaxel at 20 mg/kg (Supplementary Figure S1). Tumor formation was examined with hematoxylin&eosin staining. OVCAR-4 and OVCAR4-PR2 groups were treated for 2 months. STOSE and STOSE-PR25 were treated until animals in the control groups reached humane endpoints, which constituted 6 weeks. Thirty percent of animals in the STOSE-PR50 group were prone to developing injection site lesions, which began ulcerating on the seventh week post tumor inoculation, requiring sacrifice at an earlier time point, 5 weeks after initiation of treatment. Mice bearing OVCAR-4 were tumor-free, while those bearing OVCAR4-PR2 had residual tumors (Supplementary Figure S1). The tumor burden in the STOSE group was reduced by 86% after the paclitaxel treatment (Supplementary Figure S1). The reduction in the tumor burden after the paclitaxel treatment in both STOSE-PR25 and –PR50 groups was not statistically significant (Supplementary Figure S1). Peritoneal dissemination indices were recorded and analyzed; significant differences were observed where changes in tumor burden between treated and untreated groups were significant (Supplementary Figure S1). Overall, paclitaxel IC50 values correlated well with the in vivo drug response. Collectively, these data indicate that the pulsed-selection strategy yielded subclones of OVCAR-4 and STOSE that displayed complete or partial paclitaxel resistance in vitro and in vivo, which provided a range of models for further studies.

### 3.2. Paclitaxel Resistance in Selected Subclones Is Accompanied by Upregulation of Tau

**Figure 1:**
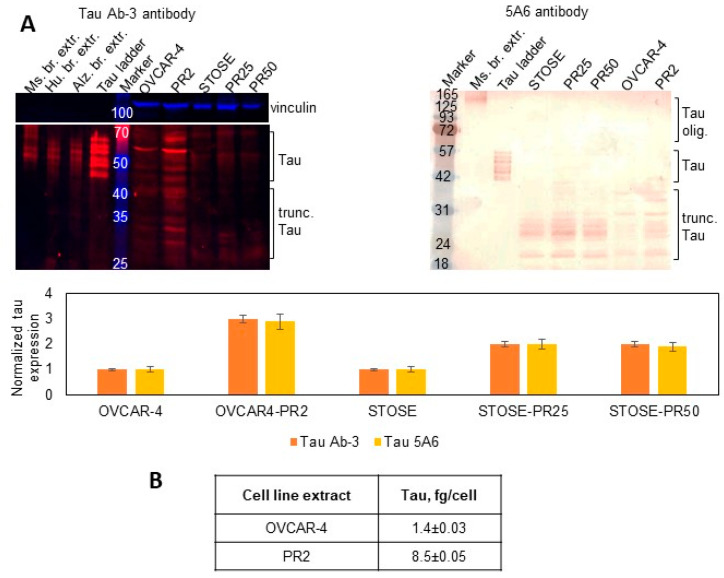
Expression of tau in parental cell lines and paclitaxel-resistant clones. (**A**) Expression of tau was examined with Western blot using tau Ab-3 antibodies (1:100 dilution) and 5A6 antibodies (1:50 dilution) in the parental and paclitaxel-resistant cells and measured with digital densitometry. Vinculin (1:1000 dilution) was used as a loading control. Mouse and human brain extracts and tau ladder consisting of 6 tau isoforms were used as positive controls. The uncropped blots are shown in File S1. (**B**) Total tau was measured by ELISA in cell extracts and derived as fg/cell.

Several clinical studies conducted in ovarian and other cancers demonstrated that response to paclitaxel treatment strongly correlated with no or low tau expression in treatment-naïve specimens, while high tau was indicative of the paclitaxel-resistant state (reviewed in Ref. [43]). Our previous studies indicated that tau was expressed in a higher number of metastatic than primary ovarian cancer specimens, and stimulation with three-dimensional collagen type I, a model of the extracellular matrix at metastatic sites in the peritoneal cavity [79,80], resulted in upregulation of tau and increased paclitaxel resistance that was reversed by the siRNA-driven tau downregulation [41]. Hence, I tested tau expression in parental cells and their paclitaxel-resistant clones. I used Tau Ab-3 antibody developed against the C-terminal region of tau and found that the total expression of tau isoforms, oligomers, and truncated tau was over two-fold higher in subclones compared to parental cell lines (Figure 1A). I also used the 5A6 tau antibody developed against the N-terminal region of the protein (amino acids 19–46) and found that the lowest molecular weight tau isoform as well as truncated tau species within the 20–40 kDa range were upregulated in paclitaxel-resistant clones (Figure 1A). I used ELISA to quantitatively measure intracellular tau concentration and found that it was increased six-fold in OVCAR4-PR2 in comparison with OVCAR-4 (Figure 1B). Collectively, these data indicate that tau was upregulated when cells gained paclitaxel resistance. Data generated by using two different antibodies with epitopes at the N- and C-terminal regions suggest that oligomer tau species and most tau isoforms are truncated at the N-terminus as they could be detected by the ab-3 tau antibody but not the 5A6 antibody. At the same time, truncated tau species and isoforms that retained the N-terminal region are also present and recognized by the 5A6 antibody. Such vast variety of tau species indicates a potential for tau multifunctionality and suggests that different molecular mechanisms could be supported by tau in ovarian cancer.

**Figure 2:**
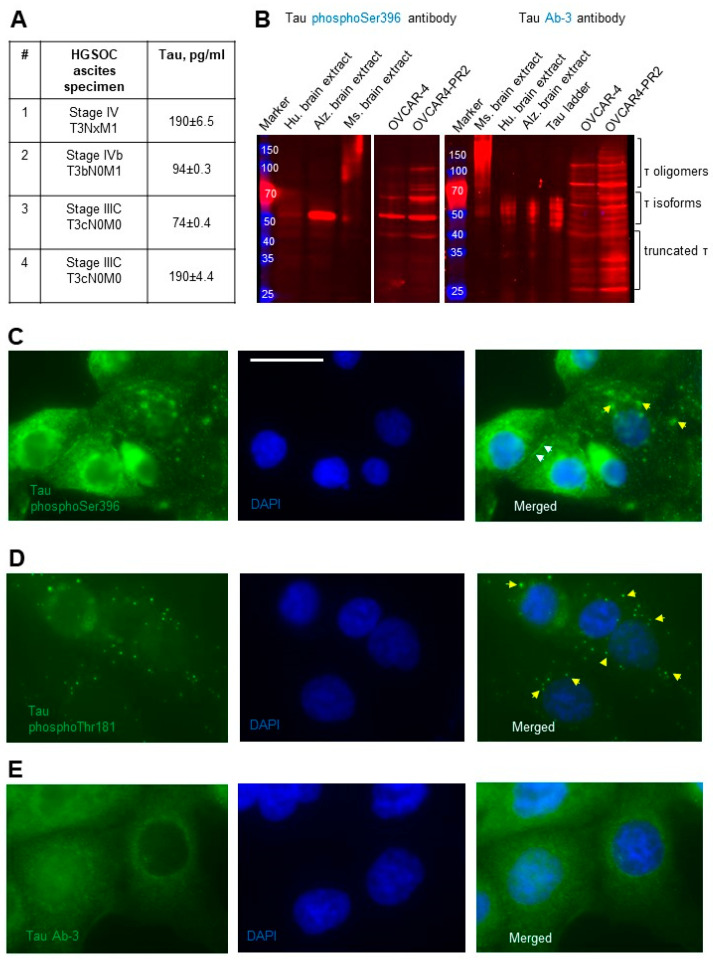
Detection of secreted and phosphorylated tau in HGSOC ascites and cell lines. (**A**) Total tau was measured by ELISA in specimens from patients with advanced and metastatic HGSOC and derived in pg/mL. (**B**) Expression of tau was examined with Western blot using tau phosphoSer396 antibodies (1:1000 dilution) and tau Ab-3 antibodies (1:100 dilution) in the parental and paclitaxel-resistant cells. Mouse and human brain extracts (20 µg/lane) and tau ladder consisting of 6 tau isoforms were used as positive controls. The loading control (vinculin) for OVCAR-4 and OVCAR4-PR2 is shown in Figure 1A. The uncropped blots are shown in File S1. (**C**) Tau phosphoSer396 primary antibodies (1:100 dilution) and anti-rabbit DyLight488 secondary antibodies were used to detect subcellular localization of the phosphoSer396 tau species in OVCAR-4 by immunofluorescence staining. Nuclear DNA was detected by staining with DAPI. Images were acquired using Zeiss fluorescence microscope. Left panel—tau (green), center panel—DAPI (blue). Right panel: images of tau and DAPI were superimposed using Zeiss Axiovert software. Bar, 25 micron. Yellow arrows point to tau in the cytoplasm, and white arrows point to tau on the cell surface. (**D**) Tau phosphoThr181 primary antibodies (1:100 dilution) and anti-mouse AlexaFluor488 secondary antibodies were used to detect subcellular localization of the tau species (green fluorescence) in OVCAR-4 with immunofluorescence staining. Nuclear DNA was detected by staining with DAPI. Images were acquired using Zeiss fluorescence microscope. Left panels—tau (green), center panels—DAPI (blue). Right panels: images of tau and DAPI were superimposed using Zeiss Axiovert software. Yellow arrows point to tau in the cytoplasm. (**E**) Tau Ab-3 primary antibodies (1:100 dilution) and anti-rabbit DyLight488 secondary antibodies were used to detect subcellular localization of tau species in OVCAR-4 by immunofluorescence staining. Nuclear DNA was detected by staining with DAPI. Images were acquired using Zeiss fluorescence microscope. Left panel—tau (green), center panel—DAPI (blue). Right panel: images of tau and DAPI were superimposed using Zeiss Axiovert software.

I measured total tau in the ascites from patients with advanced stages of HGSOC by ELISA to determine if any of the protein could be secreted outside and found that it was detectable (Figure 2A). Detection of tau in the ascites suggested that it may be secreted, and, therefore, likely hyperphosphorylated as hyperphosphorylation of tau has been associated with its ability to secrete outside the cell rather than bind to microtubules. Further, I used tau antibodies developed against phosphorylated protein residues Ser396, Thr181, and Tyr231 to determine the scope of phospho-tau expression in OVCAR-4. Using tau phosphoSer396 antibodies, I found that some (but not all) tau oligomers, isoforms, and truncated species were phosphorylated at Ser396 in OVCAR-4. Further, hyperphosphorylation of these oligomers and isoforms of tau was detected in the paclitaxel-resistant clone OVCAR4-PR2 in comparison to the parental OVCAR-4 (Figure 2B). I also examined expression of phosphoSer396 tau species using immunostaining and observed punctate and diffused cytoplasmic and cell surface expression (Figure 2C). Punctate cytoplasmic phosphoThr181 tau species were also identified with immunostaining (Figure 2D); however, no immune-reactivity with the phosphoTyr231 tau antibodies was recorded (Supplementary Figure S2). Punctate and diffuse cytoplasmic staining of total tau was detected with tau Ab-3 antibodies (Figure 2E). These data suggest the existence of mechanisms supported by phosphoSer396 and phosphoThr181 species, thus further increasing the potential complexity of tau biology in ovarian cancer.

### 3.3. Targeting Tau with Leucomethylene Blue Is Effective in Chemotherapy-Resistant Models

While the function of hyperphosphorylated tau is not known in HGSOC, the finding that it is upregulated in paclitaxel-resistant disease suggests that it may play a role in drug resistance and/or disease progression. Non-pathological microtubule-bound tau is thought to be important for cell homeostasis as it regulates microtubule assembly. Importantly, longer overall and progression-free survivals of ovarian cancer patients significantly correlated with low or no tau expression [28], indicating that reducing tau expression could extend expectancy of life for the patients. Hence, even though the role of individual tau isoforms and other tau species in HGSOC is not known, it appears that global reduction in tau could be cytotoxic. In support of this approach, depletion of tau expression with shRNAs in a model of glioblastoma significantly increased animal survival [27]. However, to elevate the translational potential of our study, I was interested in pharmacological depletion of tau in HGSOC models rather than its genomic downregulation.

**Figure 3:**
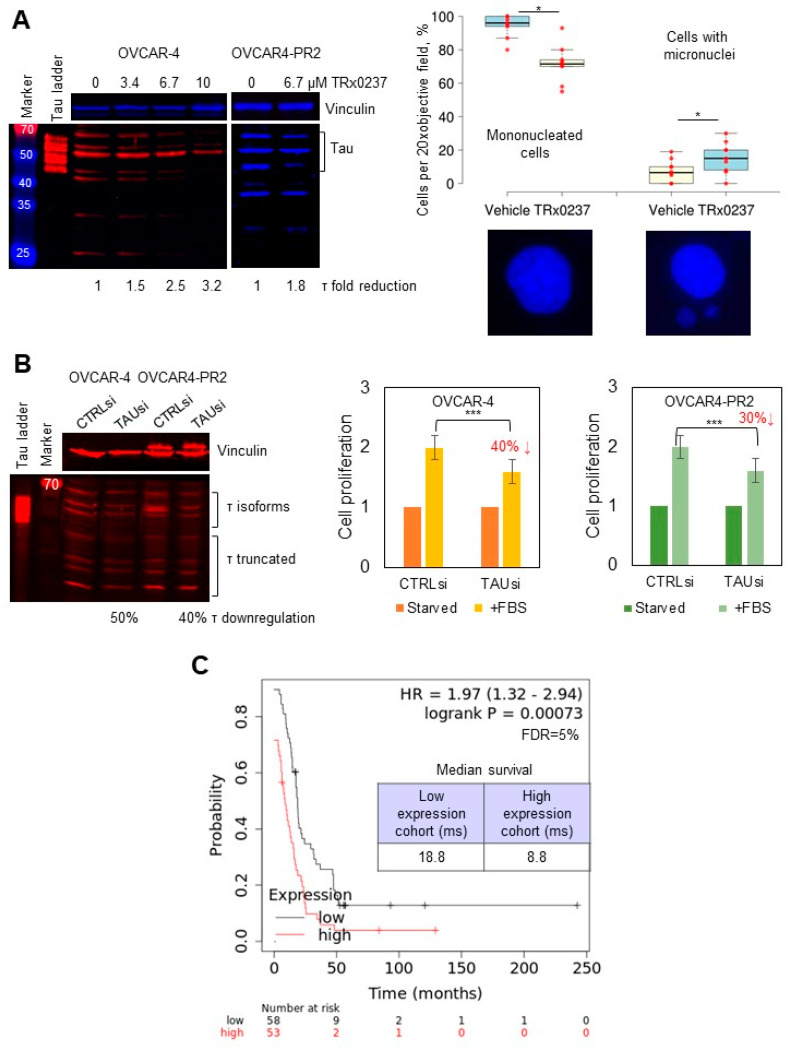
TRx0237 is cytotoxic to HGSOC cell lines, and low levels of tau reduce cell proliferation and increase patient survival. (**A**) Expression of tau was examined with Western blot using tau Ab-3 antibodies (1:1000 dilution); the parental and paclitaxel-resistant cells were treated with 0–13.4 µM TRx023 and measured with digital densitometry. Vinculin was used as a loading control. Tau ladder consisting of 6 tau isoforms was used to determine positions of tau isoforms. OVCAR-4 were treated, or not, with 6.7 µM TRx0237 for 24 h, fixed, and stained with DAPI. Cell nuclei were examined in 5 fields of view/slide in three independent experiments. The number of mononucleated cells and cells with micronuclei was quantified, averaged, derived as percent of total, and statistically analyzed using Mann–Whitney U test. Representative images are shown below the graph panel. (**B**) Expression of tau in OVCAR-4 and OVCAR4-PR2 was downregulated with tau-specific pool of siRNAs for 3 days; on day 4 after transfection, cells were either starved or stimulated for proliferation with fetal bovine serum (FBS) for 24 h, followed by WST-1 assay. Tau downregulation was examined with Western blot. Vinculin: loading control. Positions of tau isoforms and truncated fragments are indicated. Tau ladder: 6 tau isoforms commonly found in the brain. * *p* < 0.05, *** *p* < 0.01, *t*-test; average of 5 experiments. (**C**) Kaplan–Meier plotter database was used to analyze the progression-free survival (PPS) as a function of tau expression in patients with serous ovarian cancer containing TP53 mutations; data were analyzed using logrank test; the p-value, hazard ratio (HR), false discovery rate (FDR), and median survival in months in each group are shown on the graph. Median tau expression was used to separate tau-high and tau-low groups. The uncropped blots are shown in File S1.

Thus, I sought to test the effectiveness of TRx0237 against platinum- and platinum/paclitaxel-resistant cell culture and in vivo models as they both expressed tau. TRx0237 was cytotoxic to all tested cell lines (Table 1, Supplementary Figure S3), and paclitaxel resistance did not affect TRx0237 IC50 in OVCAR4-PR2 and STOSE-PR25 in comparison to these values for the parental cell lines, although TRx0237 IC50 for STOSE-PR50 doubled in comparison to the parental cell line. Treatment of OVCAR-4 and OVCAR4-PR2 with TRx0237 resulted in concentration-dependent reduction in tau expression (Figure 3A), consistent with the expected effect of this drug. At concentrations above 2 × IC50, TRx0237 treatment induced cell death. At suboptimal concentrations (1 × IC50), it resulted in significant increase in cells with micronuclei (Figure 3A), which was in line with previously reported data, which indicated that knockdown of tau results in a loss of the kinetochore fibers and micronucleation [81]. Fifty to forty percent reduction in tau expression with a pool of tau-specific siRNAs significantly reduced cell proliferation, indicating that phenothiazine-induced cytotoxicity was, to a large degree, due to tau downregulation (Figure 3B). An independent study has also reported that downregulation of tau in ovarian cancer cell lines with siRNAs resulted in reduced cell proliferation [82]. In support of the prior studies, which found that low tau correlated with better overall and progression-free survival in patients with serous ovarian cancer [28], examination of survival data for patients with serous ovarian cancer bearing TP53 mutations in a Kaplan–Meier plotter database indicated that those with low tau expression survived 10 months longer than their counterparts with high tau expression (Figure 3C) [83].

**Figure 4:**
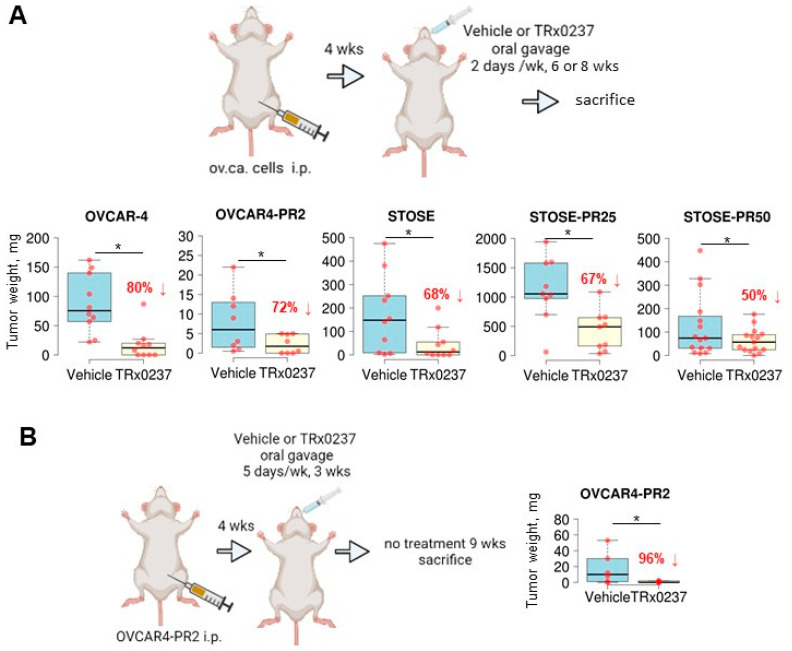
TRx0237 is effective in reducing tumor burden in parental and paclitaxel-resistant isogenic cell lines OVCAR-4 and STOSE. (**A**) The scheme represents experimental design wherein 10^6^ cells were i.p. injected into either athymic nude (OVCAR-4 n = 10/group, OVCAR4-PR2 n = 8/group) or FVB/N (STOSE n = 10/group, STOSE-PR25 n = 9/group, STOSE-PR50 n = 15/group) mice, allowed to form tumors for 2 (STOSE) or 4 (OVCAR-4) weeks, followed by the treatment with twice weekly TRx0237 at 0.5 mg/mouse or vehicle by oral gavage for 6 (STOSE) or 8 (OVCAR-4) weeks and sacrificed. (**B**) 10^6^ OVCAR4-PR2 cells were i.p. injected either athymic nude mice (n = 6/group), allowed to form tumors for 4 weeks, followed by treatment with 5 times/week TRx0237 at 1 mg/mouse or vehicle by oral gavage for 3 weeks; after that, animals were not treated for 9 weeks and sacrificed. The images were created in BioRender. The overall i.p. tumor burden for each animal in the vehicle- and TRx0237-treated groups was found by weighing the excised tumor specimens, then plotted as box plots using The BoxPlotR software [84], and statistically analyzed with Mann–Whitney U test. * *p* < 0.05. Percent of tumor burden reduction is indicated on the graphs.

To evaluate the in vivo efficacy of TRx0237 in reducing tumor burden, I tested both platinum- and platinum/paclitaxel-resistant OVCAR-4 and STOSE. Cells were i.p. injected into mice abdomens and allowed to lodge peritoneal lesions for 1 month (OVCAR-4 and OVCAR4-PR2) or 2 weeks (STOSE, STOSE-PR25, and STOSE-PR50), followed by treatment with TRx0237 (Figure 4A). An animal dose of TRx0237 of 0.5 mg/mouse, which corresponded to human equivalent dose of 125 mg/day, was delivered by oral gavage twice weekly. OVCAR-4-based groups were treated for 8 weeks, STOSE- and STOSE-PR25-based groups were treated for 6 weeks, and the STOSE-PR50 group was treated for 5 weeks. After the treatment, tumors in mouse xenografts were reduced by 80 to 50 % (Figure 4A), and the peritoneal dissemination indices were significantly reduced in treated groups (Supplementary Figure S4A), which correlated with the in vitro cytotoxicity of TRx0237 in the corresponding cell lines (Table 1) and the length of treatment. Importantly, all paclitaxel-resistant clones were sensitive to TRx0237, suggesting that acquired resistance to paclitaxel does not confer resistance to TRx0237. As tau is necessary for microtubule formation, one likely mechanism by which monotherapy affected tumor growth may be related to the failure to form microtubules during cell cycle, resulting in a variety of different cell fate outcomes from survival and formation of cells with micronuclei (at suboptimal concentrations of TRx0237) to necrosis (at concentrations above 2 × IC50). Importantly, combined use of the xenograft and syngeneic models indicates that the treatment is effective in the context of compromised and fully efficient immune systems.

To determine whether any residual tumor was left after the treatment with the mouse equivalent dose of TRx0237 corresponding to the highest dose used in human clinical trials (250 mg/day), we conducted another in vivo experiment with platinum/paclitaxel-resistant OVCAR4-PR2 cells. Tumor lesions were established i.p. for 4 weeks, followed by administration of 1 mg/mouse of TRx0237 5 days/week by oral gavage (human equivalent dose of 250 mg/day) for 3 weeks, followed by 9 weeks of drug holiday. I found that the overall tumor burden reduced by 96% (Figure 4B) and the peritoneal carcinomatosis index was significantly reduced (Supplementary Figure S4B), suggesting that the treatment with a high dose of TRx0237 monotherapy may be more efficient than the lower dose (Figure 4A) in the tested platinum/paclitaxel-resistant model; however, residual tumors still remained. Thus, TRx0237 alone may not be sufficient for complete elimination of all residual platinum/paclitaxel-resistant cancer cells.

### 3.4. A Combined Treatment with TRx0237 and Paclitaxel Results in Elimination of Tumor Burden in the Paclitaxel-Resistant Model OVCAR4-PR2

**Figure 5:**
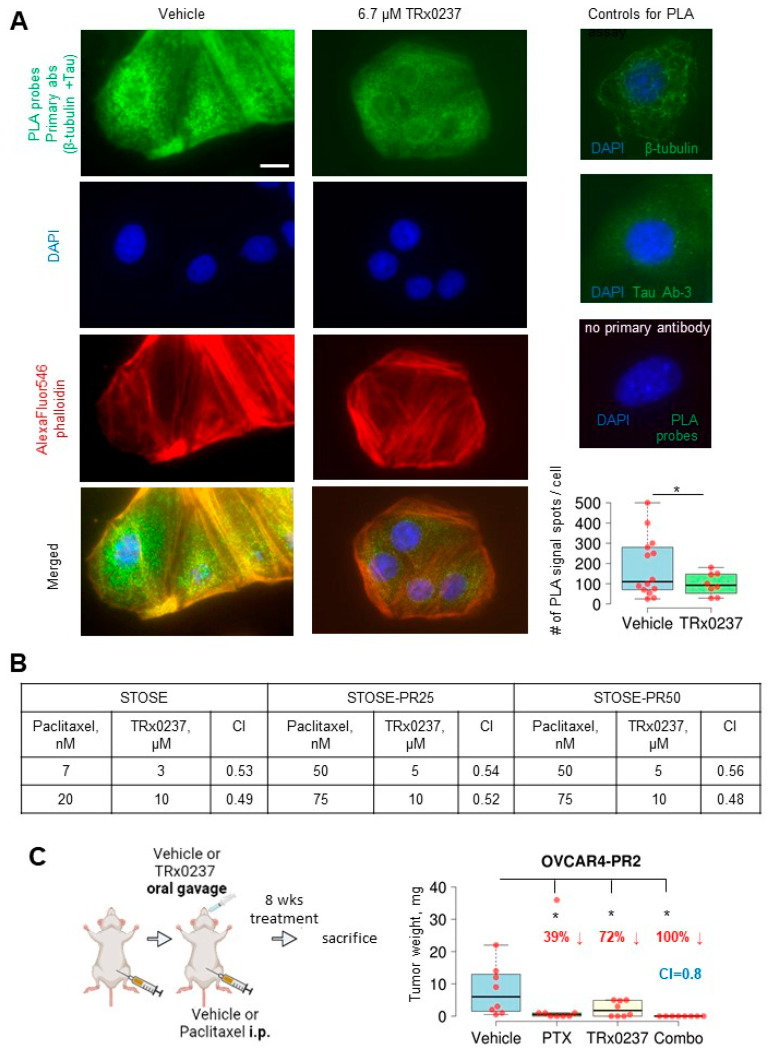
TRx0237 reduces tau bound to microtubules and synergizes with paclitaxel in eliminating tumor burden in the paclitaxel-resistant OVCAR4-PR2 xenograft model. (**A**) OVCAR-4 were treated, or not, with 6.7 µM TRx0237 for 24 h and subjected to proximity ligation assay (PLA) using tau Ab-3, β-tubulin antibodies, and green fluorescence PLA probes. The actin cytoskeleton and nuclear DNA were stained with AlexaFluor546 phalloidin and DAPI. Bar, 5 micron. Images were acquired using Zeiss fluorescence microscope. Images were superimposed using Zeiss Axiovert software. Green fluorescence signal generated due to PLA probes when both tau and β-tubulin antibodies are 0–40 nm was quantified as spots/cell in 10 randomly selected cells in three independent experiments, averaged, plotted as box graphs using The BoxPlotR software, and statistically analyzed with Mann–Whitney U test. * *p* < 0.05. Immunostaining with tau Ab-3 and β-tubulin antibodies (1:100 dilution) individually and with no primary antibodies used as controls. (**B**) STOSE and its paclitaxel-resistant clones were treated for 24 h with indicated concentrations of paclitaxel or TRx0237, or not treated, followed by examination of 1% of treated cells in a clonogenic assay. Colonies of ≥50 cells were quantified. The Combination Index (CI) was calculated based on the Chou-Talaley method and Bliss Independence model [85]. (**C**) 10^6^ OVCAR4-PR2 cells were i.p. injected in athymic nude mice (n = 8/group), allowed to form tumors for 4 weeks, and treated for 8 weeks with vehicle, twice weekly i.p. paclitaxel (PTX) at 20 mg/kg, twice weekly TRx0237 at 0.5 mg/mouse by oral gavage, and twice weekly i.p. paclitaxel (PTX) at 20 mg/kg combined with twice weekly TRx0237 at 0.5 mg/mouse by oral gavage, followed by sacrifice at the end of treatment. The images were created in BioRender. The overall i.p. tumor burden for each animal in the vehicle- and TRx0237-treated groups was found by weighing the excised tumor specimens, then plotted as box plots using The BoxPlotR software, and statistically analyzed with Mann–Whitney U test. * *p* < 0.05. Percent of tumor reduction is indicated in the graphs.

As both paclitaxel and tau can bind to the same binding site on the inner side of the microtubule, reduction in tau expression is thought to increase the number of paclitaxel binding sites that may result in resensitizing paclitaxel-resistant cells to the drug [29,42,43]. Although tau-degraders do not affect tau-tubulin interaction per se [56], reduction in levels of tau could lead to reduction in tau associated with tubulin in microtubules. To test this, I conducted a Duolink proximity ligation assay (PLA), which detects epitopes located 0-40 nm apart and can be used to detect protein–protein interaction in situ. I used PLA to determine protein–protein interaction for tau and β-tubulin in cells treated, or not, with TRx0237 and found that the amount of tau associated with β-tubulin decreased after treatment (Figure 5A). These data suggest that treatment with TRx0237 resulted in reduction in tau associated with microtubules likely due to the overall reduction in tau levels. This indicated that paclitaxel treatment may be more efficient if its binding sites are liberated as a result of reduced tau. Consistent with the idea that tau competes with paclitaxel for the binding sites on the inner side of the microtubules, resulting in reduced efficacy of the latter, combination of paclitaxel with TRx0237 synergized in reducing clonogenic ability of both parental and paclitaxel-resistant subclones of STOSE in vitro, and the combination index (CI) of the paclitaxel/TRx0237 ranged between 0.48 and 0.56, suggesting a strong to moderate level of synergy (Figure 5B). Most importantly, paclitaxel and TRx0237 synergized in achieving 100% tumor reduction in the platinum/paclitaxel-resistant OVCAR4-PR2 xenograft model (Figure 5C) and accompanied by significant reduction in peritoneal carcinomatosis index (Supplementary Figure S5). These data suggest that further development of this combination could be beneficial for significantly reducing or completely eliminating residual tumor in the platinum/paclitaxel-resistant state.

### 3.5. Mechanisms Regulating Drug Resistance Are Activated in Paclitaxel-Resistant Ovarian Cancer Cells

**Figure 6:**
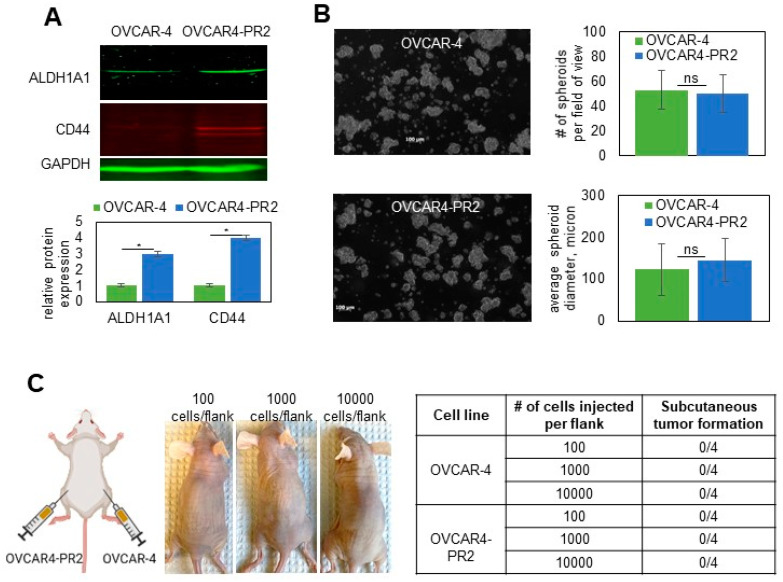
Examination of cancer-stem-cell-like characteristics of the isogenic pair of parental and paclitaxel-resistant OVCAR-4. (**A**) Expression of ALDH1A1 and CD44 in OVCAR-4 and OVCAR4-PR2 was examined with Western blot using antibodies at 1:250 and 1:500 dilution, respectively, measured with digital densitometry, plotted, and analyzed with Student’s *t*-test. * *p* < 0.05. GAPDH (1:200 dilution) was used as a loading control. The uncropped blots are shown in File S1. (**B**) OVCAR-4 and OVCAR4-PR2 (10^6^ cells/P100 tissues culture plate) were subjected to spheroid formation assay for 48 h and imaged. The number and diameter of spheroids in the field of view at 5× magnification on the objective were quantified in 10 random fields/plate in 3 independent experiments, averaged, plotted, and analyzed with Student’s *t*-test. (**C**) Scheme created in BioRender shows that OVCAR-4 were injected in the right flanks, and OVCAR4-PR2 were injected in the left flanks at 100 cells/flank, 1000 cells/flank, and 10,000 cells/flank and observed for tumor formation for 15 months. Images of mice after 15 months of observation demonstrate that animals did not develop subcutaneous tumor nodules by the end of the experiment, which is reflected in Table 1.

As drug resistance is a complex state regulated by many diverse mechanisms, we wished to uncover some of those that, in addition to tau, may regulate paclitaxel resistance. To address this, I focused on OVCAR4-PR2 as it is a human-derived cell line that contains a clinically relevant mutation in TP53 (Leu130Val) [72] and replicates the pattern of metastatic colonization in the peritoneal cavity [74,75]; hence, it could serve as a reliable model to study recurrent, platinum/paclitaxel-resistant disease. It was previously reported that ALDH1A1 was upregulated in a drug-tolerant subpopulation of cancer [86]. I also observed upregulation of ALDH1A1 in OVCAR4-PR2 in comparison with OVCAR-4 (Figure 6A). As ALDH1A1 is also a cancer stem cell marker in ovarian cancer [87], I examined whether acquisition of paclitaxel resistance is accompanied by development of the cancer stem cell phenotype. I found that CD44, another cancer stem cell marker [88], was upregulated in OVCAR4-PR2 as well (Figure 6A). However, the acquisition of paclitaxel resistance and upregulation of CD44 did not improve upon the spheroid-forming ability of OVCAR-4 (Figure 6B) as the number and size of spheroids did not significantly differ among the two cell lines. Further, I examined the in vivo self-renewal capacity of the paclitaxel-resistant clone in comparison with that of the parental cell line in a mouse model. I injected a limited number of cells (100–10,000 of either OVCAR-4 or OVCAR4-PR2 cells/flank subcutaneously) to test the ability of these cells to form tumors. We did not observe tumor formation in both conditions for as long as 15 months (Figure 6C). These data indicate that, although OVCAR4-PR2 is resistant to platinum and paclitaxel and is characterized by upregulation of drug resistance and cancer stem cell markers, such as tau, ALDH1A1, and CD44, it did not gain a true cancer stem cell phenotype in the process of its transformation. However, as the paclitaxel-resistant cell line was derived in vitro, a possibility remains that it did not capture all possible features associated with drug resistance, including stemness.

As OVCAR-4 was derived from ascites of a cisplatin-refractory HGSOC patient, this cell line displayed the IC50 for carboplatin in the submillimolar range (0.1 mM, Supplementary Table S1). Acquisition of paclitaxel resistance in OVCAR4-PR2 did not associate with increased resistance to carboplatin (Supplementary Table S1), suggesting that mechanisms regulating paclitaxel resistance in this cell line do not affect mechanisms of DNA damage repair.

Altogether, these data are consistent with the expected molecular and phenotypical changes rendered by resistance to paclitaxel but not acquisition of multidrug resistance (as it applies to carboplatin or TRx0237) or the cancer stem cell phenotype.

### 3.6. Examination of Pathways Dysregulated in OVCAR-4 after Acquisition of Paclitaxel Resistance

**Figure 7:**
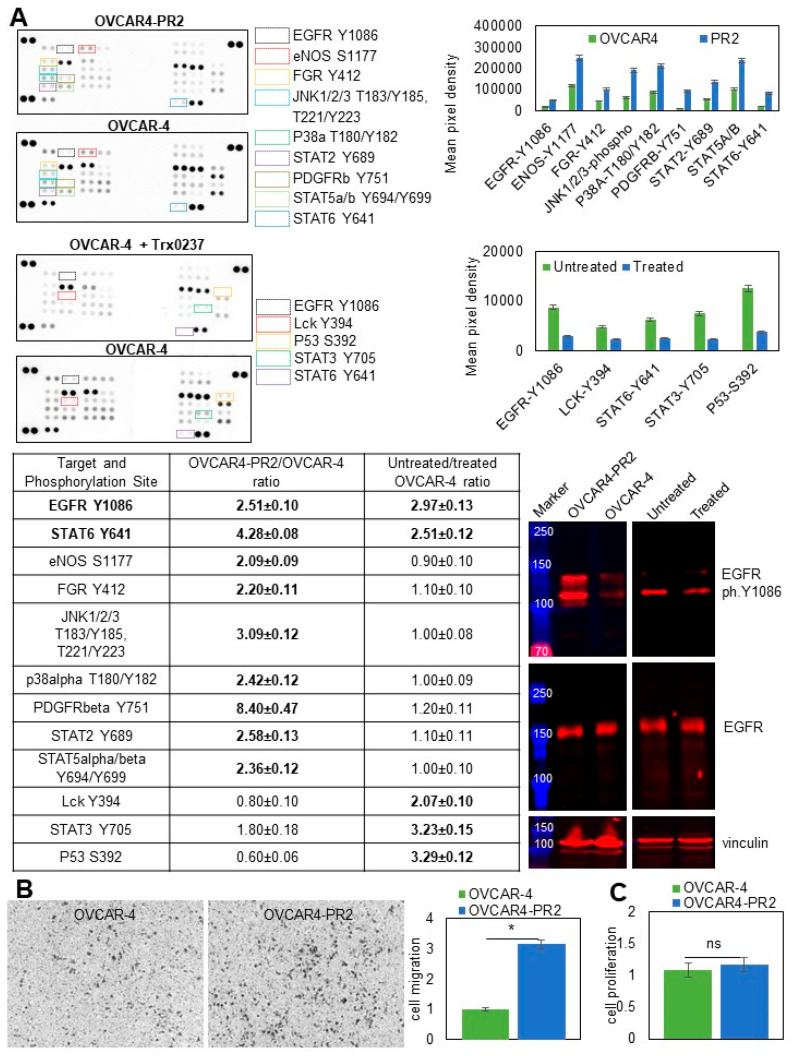
Paclitaxel-resistant phenotype is associated with changes in kinase activation that are partially reversed by the treatment with TRx0237. (**A**) Human phospho-kinase antibody array was used to determine relative levels of phosphorylation in 37 kinases and 2 related proteins in OVCAR-4 and OVCAR4-PR2, as well as OVCAR-4 treated with 6.7 µM TRx0237 for 72 h and untreated OVCAR-4. Mean pixel density was measured by digital densitometry and Azure Biosystems software and plotted on the graphs. Mean pixel density in OVCAR4-PR2 was divided by that in OVCAR-4, and mean pixel density in OVCAR4 was divided by that in OVCAR-4 treated with TRx0237. Ratios > 2 were considered biologically significant and are shown in Table 1. Expression of phosphoY1086-EGFR (1:5000 dilution) and EGFR (1:200 dilution) was examined with Western blot. The uncropped blots are shown in File S1. (**B**) 10^5^ cells were seeded atop Transwell inserts with 8-micron pores that were coated with collagen type I on the bottom and allowed to migrate for 9 h. Non-migrated cells were removed; migrated cells were fixed, stained, imaged with Zeiss Axiovert software, counted, plotted, and statistically analyzed with Student’s *t*-test. * *p* < 0.05. (**C**) Cell proliferation was measured using the WST-1 assay. OD440 values for the parental OVCAR-4 were arbitrarily set at 1, and the corresponding values for OVCAR4-PR2 were calculated accordingly, plotted, and statistically analyzed with Mann–Whitney U test.

Receptor tyrosine and cytoplasmic kinases are frequently aberrantly activated in cancers, and their function has been linked to development of a paclitaxel-resistant phenotype [89,90,91]. To determine whether gaining paclitaxel resistance in platinum-resistant cells was accompanied by sustained dysregulation of several pleotropic genes commonly associated with cancer progression, I conducted a focused proteomics study. I found that EGFR, eNOS, FGR, JNK1/2/3, p38α, PDGFRβ, STAT2, STAT5α/β, and STAT6 were over-activated in the platinum/paclitaxel-resistant cells in comparison to the parental platinum-resistant ones (Figure 7A). I then examined the status of these pathways in OVCAR-4 that were treated, or not, with TRx0237 at 1 × IC50 concentration and observed increased phosphorylation of EGFR at Y1086, Lck at Y394, TP53 at S392, STAT3 at Y705, and STAT6 at Y641 in the untreated cells. This suggested that acquisition of paclitaxel resistance and treatment with TRx0237 resulted in activation of several proteins; notably, changes in the activation status of EGFR and STAT6 were detected in both experimental conditions (Figure 7A). Activation of EGFR in OVCAR4-PR2 over OVCAR-4 and inactivation in OVCAR-4 treated with TRx0237 in comparison with the untreated controls was examined and confirmed with Western blot (Figure 7A). Thus, this study indicated that this treatment can partially reverse over-activation of kinases associated with the paclitaxel-resistant phenotype.

Receptor tyrosine and mitogen-activated protein kinases found to be activated in paclitaxel-resistant cells have been previously linked to cell migration, one of the essential functions of metastatic cells [92,93,94,95]. Using a Transwell migration assay, I found that migration of OVCAR4-PR2 increased by over three-fold compared to OVCAR-4 (Figure 7B). Increased cell migration in OVCAR4-PR2 was not due to changes in cell proliferation (Figure 7C). These data suggest that activation of both selected kinase pathways may have contributed to a phenotypical functional change in cell migration. Likewise, as with EGFR, JNK, p38α, PDGFRβ, STAT2, and STAT5α/β are pleotropic genes, and their activation could contribute to a drug-resistant phenotype by activating a multitude of signaling pathways and facilitating other essential functions of a metastatic cell that remain to be investigated.

## 4. Discussion

Chemotherapy resistance in high-grade serous tubo-ovarian carcinoma constitutes a terminal stage of the disease and is responsible for the mortality associated with this malignancy. This study suggests that targeting tau in platinum- and platinum/paclitaxel-resistant HGSOC may be a viable approach to reducing tumor burden and increasing survival. To this end, it is important to extend examination of the efficacy of TRx0237 to other human-derived models of HGSOC in the future. Based on the findings that tau is important regarding formation of long labile microtubule domains and its depletion results in an increase in stable microtubule domains [26], it is likely that removal of tau by degradation with phenothiazines leads to blocking of the cell cycle due to disruption in microtubule dynamics. Further, reduction in tau with phenothiazines could liberate paclitaxel binding sites on the microtubule, which may have rendered paclitaxel treatment more effective.

This study suggests that tau in HGSOC is expressed in a wide range of possible forms, such as oligomers or big tau, six isoforms originally discovered in the brain, and truncated species. We have previously reported on intracellular tau located in the cytoplasm and the nucleus in cell lines and tissues from patients [41]; in this study, I add evidence for the existence of extracellular tau as well as hyperphosphorylated tau. These findings raise many questions related to the function of this protein in HGSOC. In parallel, a previous study in prostate cancer also reported on expression of several tau isoforms and their intracellular distribution and raised the possibility of tau multifunctionality [52]. In speculating about the role of different forms of tau in HGSOC, one important consideration is the tau domain composition [24,43,96,97,98]. Although it remains to be established to what extent the basic function of different tau proteins in other cell types applies to HGSOC, many biochemical studies have uncovered functional roles of tau domains in its binding to microtubules and self-assembly. A study of endogenous N-terminally truncated tau species suggested that this region is directly involved in stabilizing microtubules [99]. Interestingly, while the full-length tau and its fragment, including amino acids 1 to 230, had anti-apoptotic function in neuronal cells, an N-terminal fragment 1–44 induced neuronal cell death by apoptosis [100]. A short region of N-terminal tau domain between residues 15 and 28 is thought to be responsible for the protein secretion outside the cell [101]. It has been found that a truncated N-terminal fragment with the apparent molecular weight of 20–22 kDa is capable of inducing synaptic dysfunction and neurodegeneration [102]. It would be challenging to rationalize extrapolating these data on HGSOC cells as processes of survival and apoptosis are impaired in cancer cells compared to normal cells. However, mere evidence for a pathology-inducing role in N-terminal tau species in neurodegenerative diseases is suggestive that it could play a functional role in HGSOC, although more research is needed to identify whether this role would be cancer-promoting or not. Differential expression of full-size isoforms containing either three or four microtubule-binding domains can also result in diverse functional outcomes. Further, 3R tau isoforms are predominant in brain development, and 4R isoforms increase their expression throughout life, reaching equal levels with 3R isoforms in adulthood [103]. From the standpoint of disease initiation and progression, expression of 3R isoforms in Drosophila melanogaster were linked with disrupted axonal transport, locomotor deficiencies, and shorter lifespan, while expression of 4R isoforms resulted in neurodegeneration and learning deficits [104]. A shift toward an increase in 4R tau isoforms in mice resulted in increased tau phosphorylation and induced severe seizures [105]. In adult neurons, 4R tau stabilized MTs significantly more strongly than 3R tau [106], and 4R tau isoforms were demonstrated to regulate microtubule dynamics as human SH-SY5Y neuroblastoma cells with overexpressed 4R tau were characterized by an increased number of microtubules [107]. These data indicate the importance of further studies on the normal and pathological roles of 3R and 4R tau isoforms in HGSOC; however, the differential role of 3R and 4R in microtubule dynamics suggests that targeting 4R isoforms could result in disruption of the cell cycle and cancer cell death. Recent solid-state NMR spectroscopy studies along with earlier data generated with truncated tau protein suggested that the C-terminal domain of tau may prevent its self-aggregation and formation of fibrils similar to those in patients with neurodegenerative diseases [108,109]. Studies of truncated tau protein suggested that the C-terminal region is responsible for binding to the outside of the microtubules [110]; if this mechanism pertains to HGSOC, these data indicate that tau species containing C-terminal domains are valid targets for disruption of the cell cycle and inducing cancer cell death. With respect to tau hyperphosphorylation, previous reports indicated that hyperphosphorylation at Thr181, Thr231, and Ser396, among other residues, correlated with stages of fetal development [111]. It has been suggested that the process of ovarian carcinogenesis involves dedifferentiation from a well- to a poorly differentiated tumor [112], which could be accompanied by formation of cancer stem cells [113]. The data presented here indicate that acquisition of paclitaxel resistance was accompanied by acquisition of some features representing the stem cell phenotype, such as upregulation of CD44 and ALDH1A1 and hyperphosphorylation of Ser396 or tau; however, cells that acquired paclitaxel resistance did not gain self-replicating ability and did not gain phosphorylation of Thr181. Thus, it would be interesting to examine whether hyperphosphorylation of all tau residues during fetal development mimics that in cancer stem cells and whether hyperphosphorylated tau species could serve as additional cancer stem cell markers. This study indicates that escalation of drug resistance, i.e., acquisition of paclitaxel resistance in platinum-resistant cells, is accompanied by hyperphosphorylation. Hence, further studies into the patterns of tau phosphorylation could potentially lead to development of markers of disease progression. More studies are required to establish the role of secreted tau in ovarian cancer pathology and whether levels of secreted tau correlate with disease progression. Levels of total tau in specimens of cerebrospinal fluid from patients with neurodegenerative diseases ranged 17.2–1100 pg/mL [114], and this study identified total tau in ascites within the same range of concentrations, suggesting a role in pathology. It is likely that each form of tau contributes in a unique way to survival and proliferation of HGSOC cells as its global reduction by either genomic (siRNA) or pharmacological (tau degradation) approaches significantly impacted cell viability, proliferation, and tumor formation.

Mechanisms of drug resistance are complex and often dependent on many underlying mechanisms. Paclitaxel resistance due to upregulation of tau is one of these mechanisms. Interestingly, the findings presented here suggest that the clones selected based on their resistance to paclitaxel were also characterized by upregulation of tau. However, the data also indicate that tau upregulation may not be the only mechanism of paclitaxel resistance. This is especially apparent when comparing paclitaxel-resistant clones of OVCAR-4 and STOSE. While tau expression in STOSE and its clones was lower than that in OVCAR-4 and its clones, the former displayed five-fold higher IC50 for TRx0237. TRx0237 IC50 for STOSE-PR50 was twice higher in comparison with the parental STOSE and another paclitaxel-resistant clone, STOSE-PR25, which reflected in only a 50% tumor reduction after mice bearing STOSE-PR50 were treated with TRx0237 as opposed to nearly 70% tumor reduction in STOSE and STOSE-PR25. Of note, STOSE was originally more resistant to paclitaxel than OVCAR-4 and displayed nearly 20-fold higher paclitaxel IC50, suggesting pre-existing intrinsic resistance; this and upregulation of other factors may have resulted in slightly reduced efficiency of TRx0237 in STOSE models. Differential expression of tau may suggest one potential underlying mechanism; other microtubule-associated proteins, including MAP6, could execute the function played by tau [26], which would render tau downregulation less relevant in inducing cytotoxicity via disrupted microtubule assembly. This study further highlights the need for further investigation into mechanisms of drug resistance in HGSOC in order to better understand the contribution of different factors in inter- and intra-tumoral resistance.

Being a multifactorial process, acquisition of paclitaxel resistance resulted in sustained dysregulation of several cancer-related pathways, including upregulation of EGFR. The fact that these focused and limited-scope gene expression studies have uncovered significant changes indicates the importance of conducting additional high-throughput unbiased gene expression studies to better understand the full range of changes occurring when cells acquire resistance to paclitaxel. As paclitaxel is a main drug of choice in treatment of many solid malignancies, these findings could potentially apply to other cancers resistant to paclitaxel. Interestingly, treatment with TRx0237 decreased EGFR activation. It remains to be investigated whether gain of tau is mechanistically linked with activation of EGFR and whether tau degradation by TRx0237 regulates inactivation of EGFR, or whether EGFR and tau are regulated separately in paclitaxel-resistant cells, but these data suggest a possible connection of these major pathways regulating human pathologies, which would require further investigation. For example, it would be important to determine whether acquisition of the paclitaxel-resistant phenotype is associated with an increase in expression of EGFR ligands.

Barring a lack of understanding of the role of each tau form in HGSOC, the studies presented here indicate that the overall reduction in tau levels alone and in combination with paclitaxel is detrimental to cancer cells. Hence, in future studies, it would be important to uncover the underlying mechanisms by which loss of tau results in cancer cell death.

## 5. Conclusions

I demonstrate that full-length, truncated, and phosphorylated species of microtubule-associated protein tau are expressed in models of platinum/paclitaxel-resistant high-grade serous ovarian carcinoma. Importantly, I show that targeting tau with a pharmacological inhibitor, leucomethylene blue (TRx0237), effectively reduces the tumor burden in xenograft models. Finally, I report that TRx0237 synergizes with paclitaxel in eliminating platinum/paclitaxel-resistant cells in vitro and in vivo.

## Figures and Tables

**Table 1:** IC50 for TRx0237 ^1^.

Cell Line	IC50 TRx0237, µM
OVCAR-4	6.7 ± 0.2
STOSE	29.0 ± 2.1
OVCAR4-PR2	8.6 ± 0.9
STOSE-PR25	36.6 ± 3.2
STOSE-PR50	60.2 ± 6.3

^1^ The data are an average of 4 independent experiments conducted with 8 replicas ± standard deviation.

## Data Availability

The data presented in this study are available in this article and Supplementary Material.

## Supplementary Materials

The following are available online at https://www.mdpi.com/article/10.3390/cancers14184535/s1, Figure S1: Paclitaxel-challenged isogenic OVCAR-4 and STOSE cell lines display resistance to paclitaxel and upregulation of tau expression, Figure S2: Detection of secreted and phosphorylated tau in HGSOC ascites and cell lines, Figure S3: IC50 for TRx0237, Figure S4: Peritoneal dissemination index demonstrates the number of lesions within the peritoneal cavity in each condition as detailed in Figure 4A,B, Figure S5: Peritoneal dissemination index demonstrates the number of lesions within the peritoneal cavity in each condition as detailed in Figure 5, Table S1: IC50 for paclitaxel and carboplatin, Table S2: Clinicopathological human ovarian carcinoma ascites specimen data, File S1: uncropped WB images.
