# Supplementary material for: Targeting Microtubule-Associated Protein Tau in Chemotherapy-Resistant Models of High-Grade Serous Ovarian Carcinoma
Source: Cancers (Basel). 2022 Sep 19;14(18):4535. doi: 10.3390/cancers14184535 (PMC9496900; doi:10.3390/cancers14184535)
Supplement: Supplementary file 1 [file cancers-14-04535-s001.zip › File S1-Western blots - revision 2.pptx]

## Slide 1
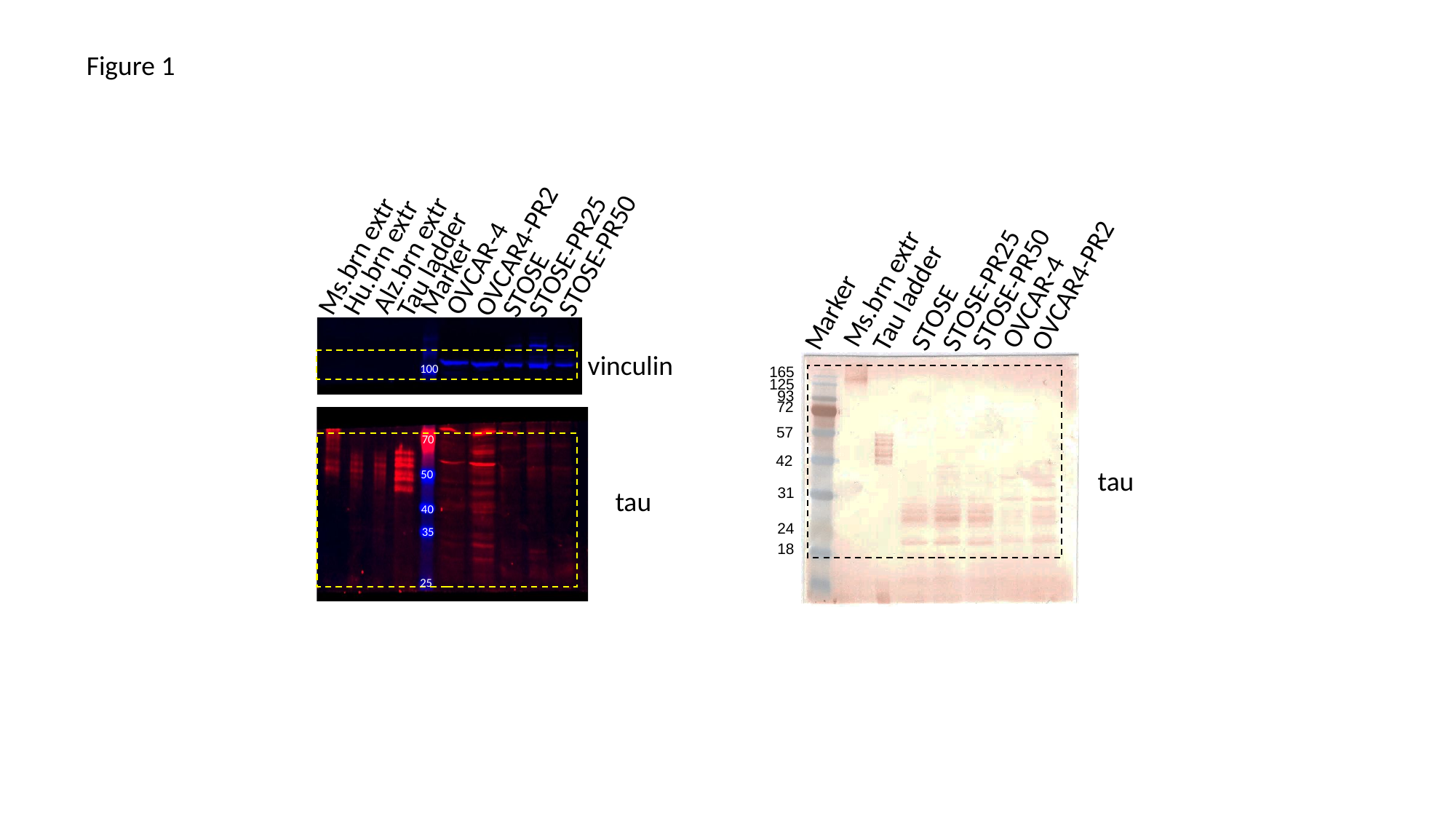

Figure 1
Alz.brn extr
Marker
OVCAR-4
Ms.brn extr
Hu.brn extr
STOSE-PR50
OVCAR4-PR2
Tau ladder
STOSE
STOSE-PR25
vinculin
tau
100
70
50
40
35
25
Ms.brn extr
OVCAR-4
Marker
STOSE-PR50
OVCAR4-PR2
STOSE
Tau ladder
STOSE-PR25
tau
165
125
93
72
57
42
31
24
18

## Slide 2
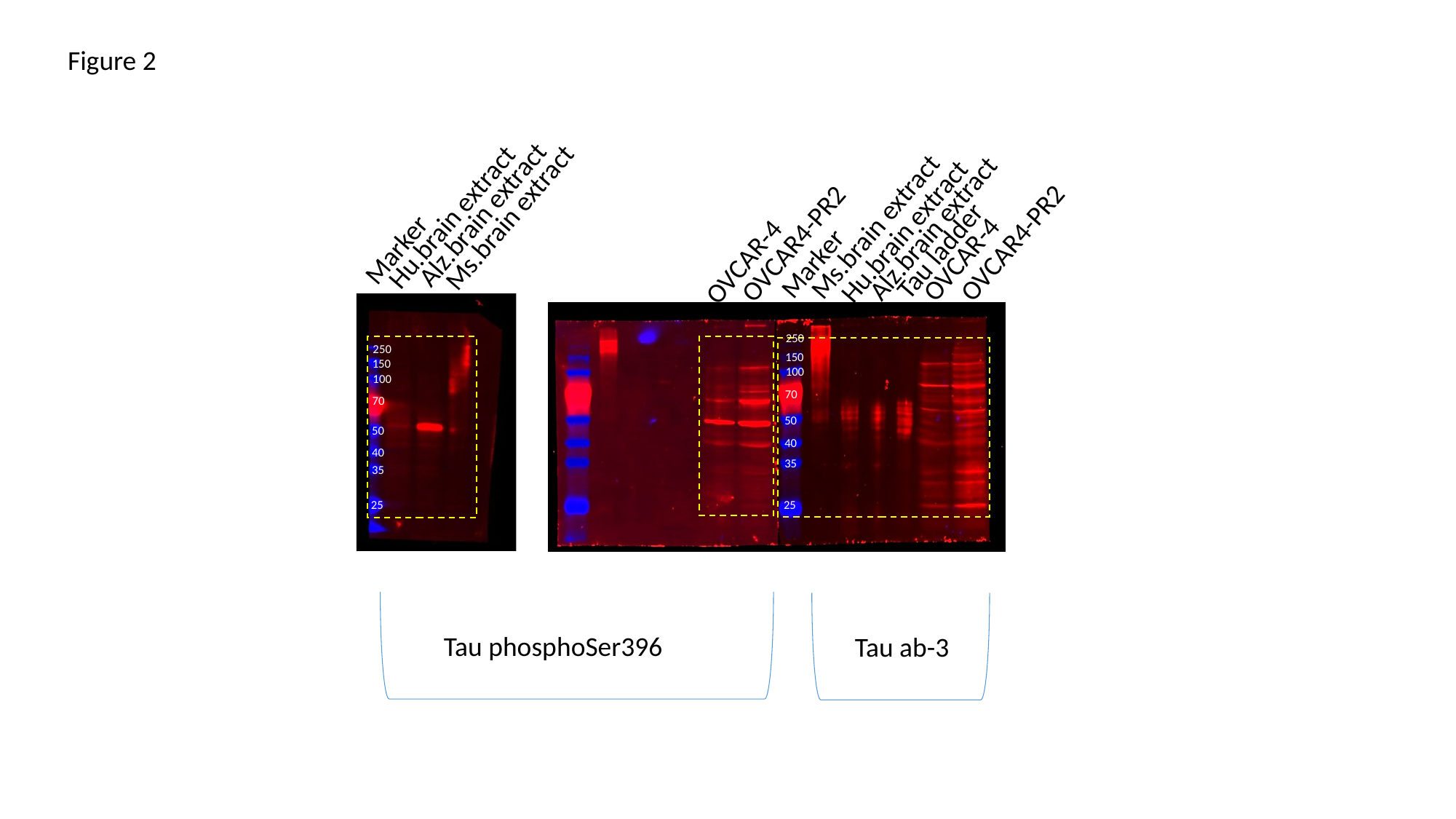

Figure 2
Marker
Alz.brain extract
Hu.brain extract
Ms.brain extract
Marker
Ms.brain extract
Alz.brain extract
Hu.brain extract
Tau ladder
OVCAR4-PR2
OVCAR-4
OVCAR4-PR2
OVCAR-4
Tau phosphoSer396
Tau ab-3
250
250
150
150
100
100
70
70
50
50
40
40
35
35
25
25

## Slide 3
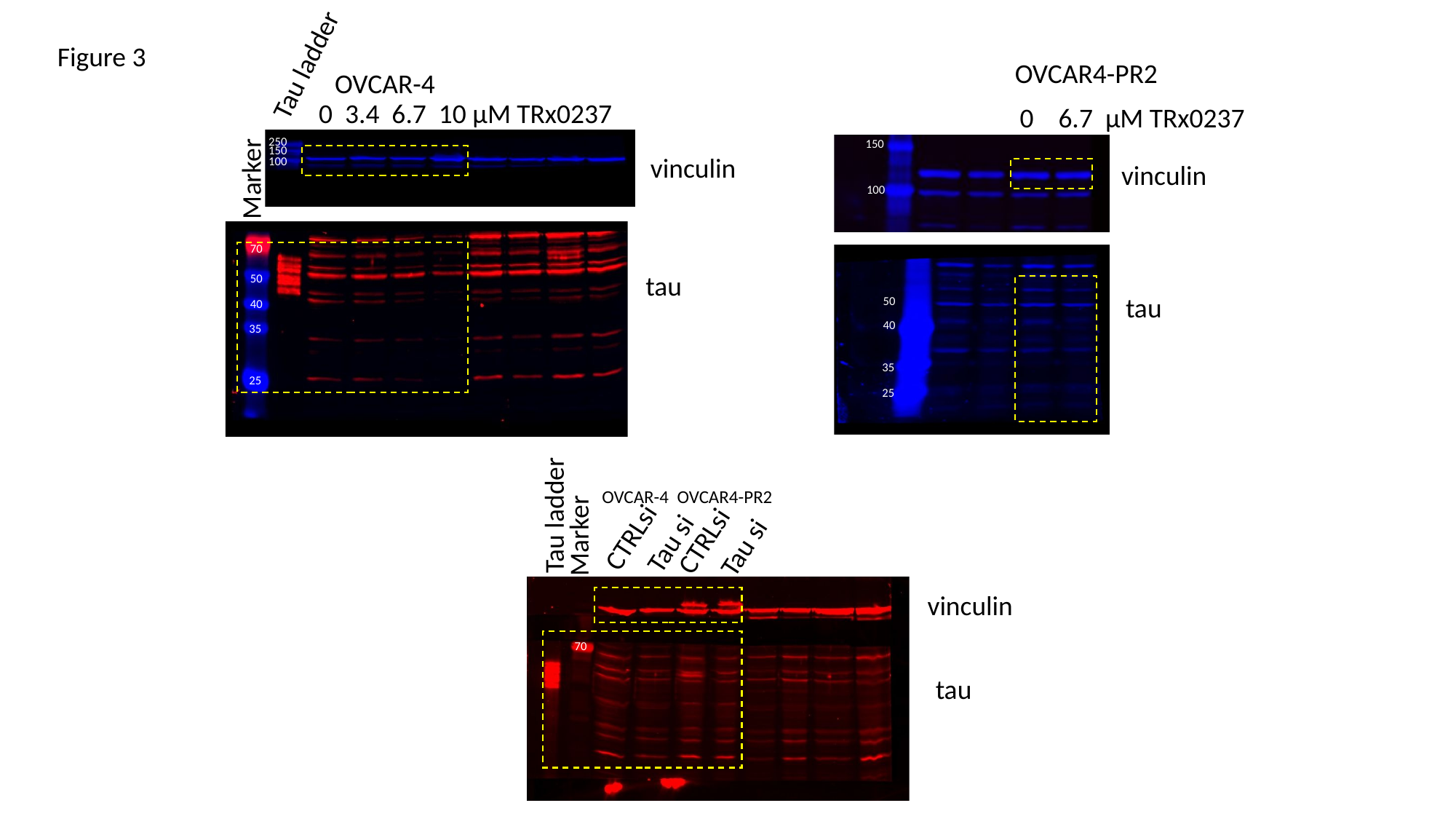

Tau ladder
OVCAR-4
0 3.4 6.7 10 µM TRx0237
Marker
vinculin
tau
250
150
100
70
50
40
35
25
Figure 3
OVCAR4-PR2
0 6.7 µM TRx0237
vinculin
tau
150
100
50
40
35
25
OVCAR-4 OVCAR4-PR2
Tau ladder
Marker
CTRLsi
Tau si
CTRLsi
Tau si
vinculin
tau
70

## Slide 4
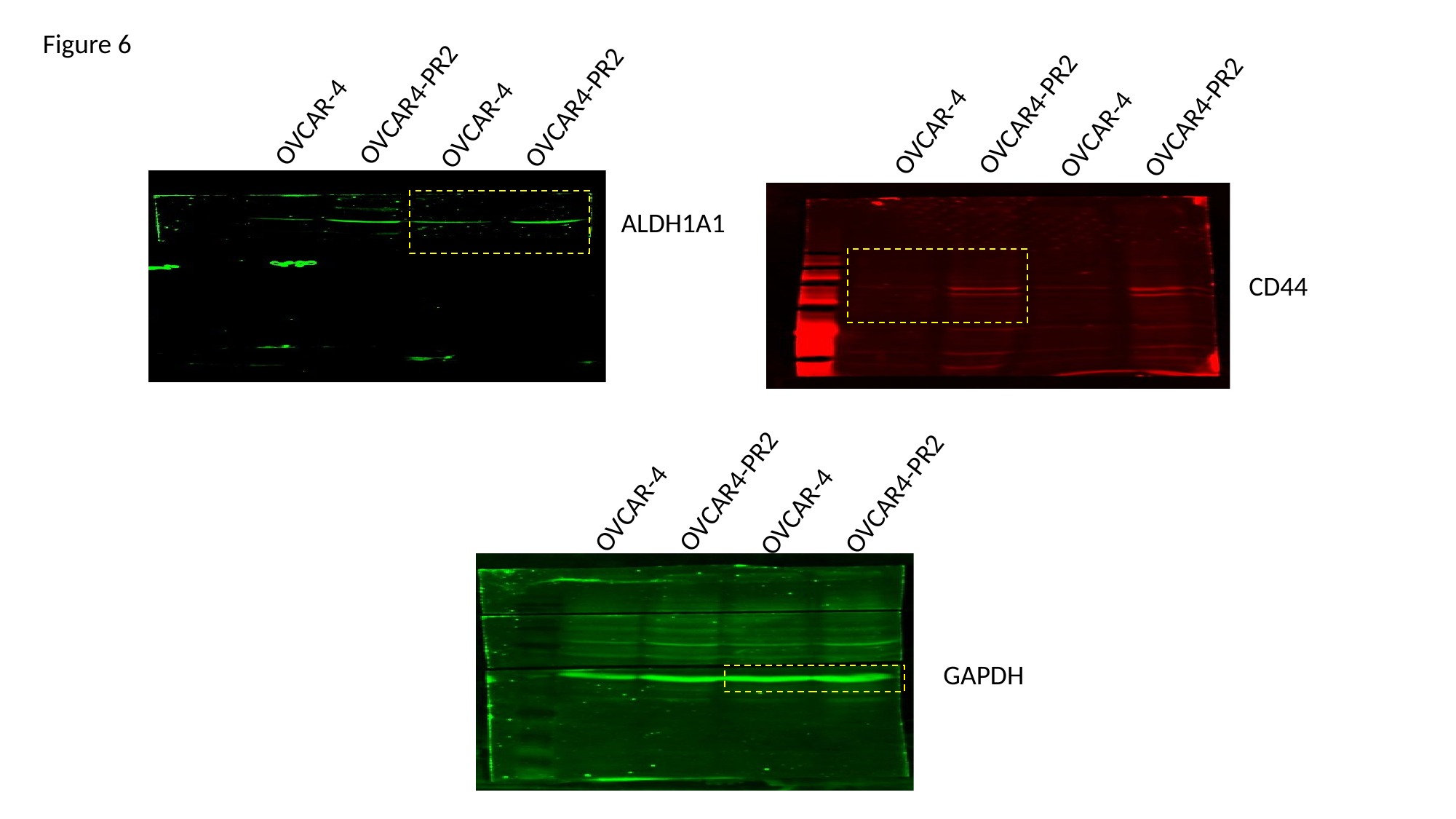

OVCAR4-PR2
OVCAR-4
OVCAR4-PR2
OVCAR-4
ALDH1A1
OVCAR4-PR2
OVCAR-4
OVCAR4-PR2
OVCAR-4
CD44
Figure 6
OVCAR4-PR2
OVCAR-4
OVCAR4-PR2
OVCAR-4
GAPDH

## Slide 5
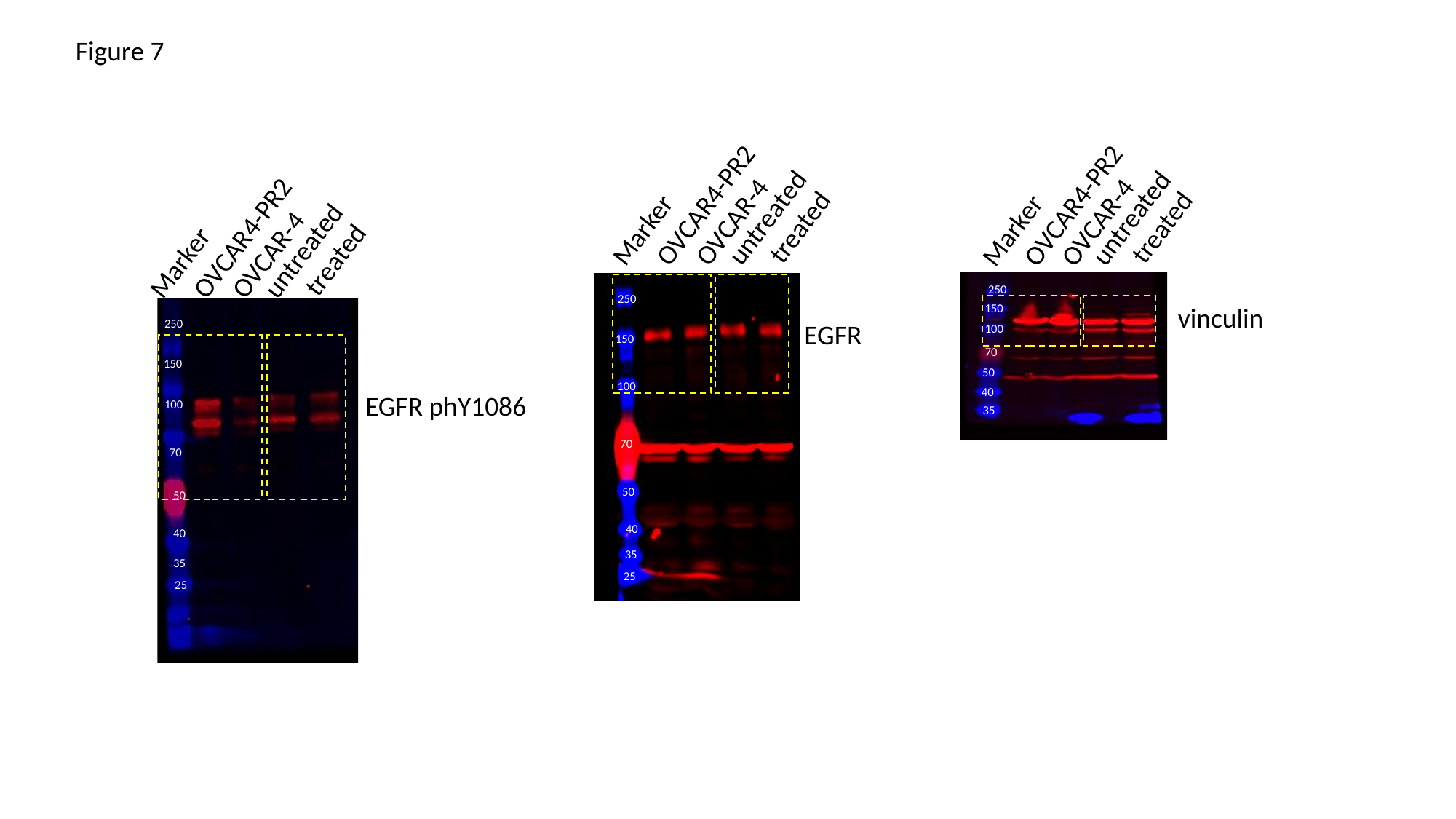

Figure 7
treated
untreated
OVCAR-4
OVCAR4-PR2
Marker
vinculin
250
150
100
70
50
40
35
treated
untreated
OVCAR-4
OVCAR4-PR2
Marker
EGFR
250
150
100
70
50
40
35
25
treated
untreated
OVCAR-4
OVCAR4-PR2
Marker
EGFR phY1086
250
150
100
70
50
40
35
25
