# Supplementary material for: Targeting Microtubule-Associated Protein Tau in Chemotherapy-Resistant Models of High-Grade Serous Ovarian Carcinoma
Source: Cancers (Basel). 2022 Sep 19;14(18):4535. doi: 10.3390/cancers14184535 (PMC9496900; doi:10.3390/cancers14184535)
Supplement: Supplementary file 1 [file cancers-14-04535-s001.zip › Supplementary Figure S1.pptx]

## Slide 1
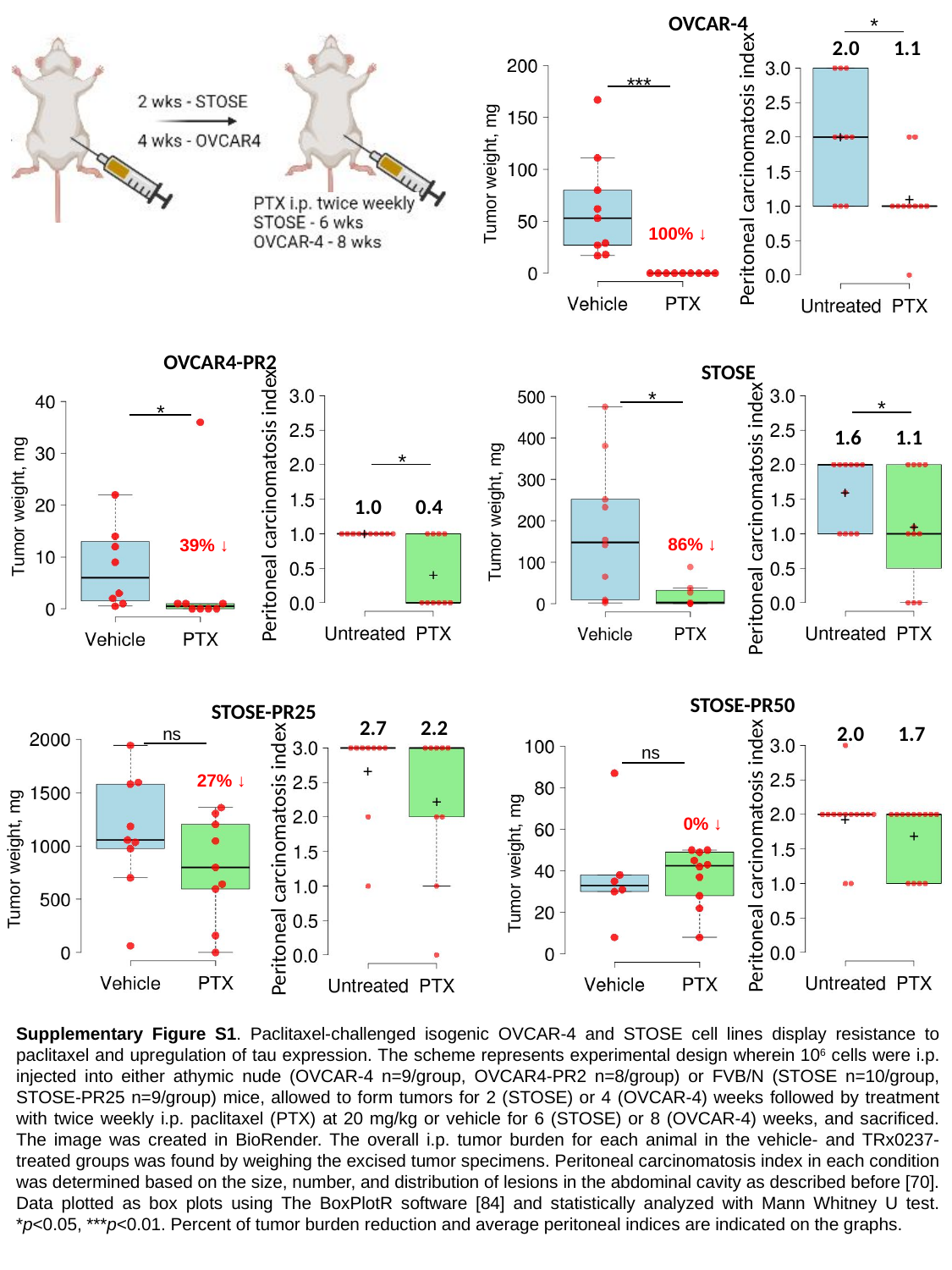

OVCAR-4
*
2.0 1.1
Peritoneal carcinomatosis index
***
Tumor weight, mg
100% ↓
OVCAR4-PR2
Peritoneal carcinomatosis index
1.0 0.4
*
39% ↓
Tumor weight, mg
*
STOSE
*
Tumor weight, mg
86% ↓
*
1.6 1.1
Peritoneal carcinomatosis index
STOSE-PR50
2.0 1.7
ns
0% ↓
Tumor weight, mg
Peritoneal carcinomatosis index
STOSE-PR25
2.7 2.2
ns
27% ↓
Tumor weight, mg
Peritoneal carcinomatosis index
Supplementary Figure S1. Paclitaxel-challenged isogenic OVCAR-4 and STOSE cell lines display resistance to paclitaxel and upregulation of tau expression. The scheme represents experimental design wherein 106 cells were i.p. injected into either athymic nude (OVCAR-4 n=9/group, OVCAR4-PR2 n=8/group) or FVB/N (STOSE n=10/group, STOSE-PR25 n=9/group) mice, allowed to form tumors for 2 (STOSE) or 4 (OVCAR-4) weeks followed by treatment with twice weekly i.p. paclitaxel (PTX) at 20 mg/kg or vehicle for 6 (STOSE) or 8 (OVCAR-4) weeks, and sacrificed. The image was created in BioRender. The overall i.p. tumor burden for each animal in the vehicle- and TRx0237-treated groups was found by weighing the excised tumor specimens. Peritoneal carcinomatosis index in each condition was determined based on the size, number, and distribution of lesions in the abdominal cavity as described before [70]. Data plotted as box plots using The BoxPlotR software [84] and statistically analyzed with Mann Whitney U test. *p<0.05, ***p<0.01. Percent of tumor burden reduction and average peritoneal indices are indicated on the graphs.
