# Supplementary material for: Targeting Microtubule-Associated Protein Tau in Chemotherapy-Resistant Models of High-Grade Serous Ovarian Carcinoma
Source: Cancers (Basel). 2022 Sep 19;14(18):4535. doi: 10.3390/cancers14184535 (PMC9496900; doi:10.3390/cancers14184535)
Supplement: Supplementary file 1 [file cancers-14-04535-s001.zip › Supplementary Figure S2.pptx]

## Slide 1
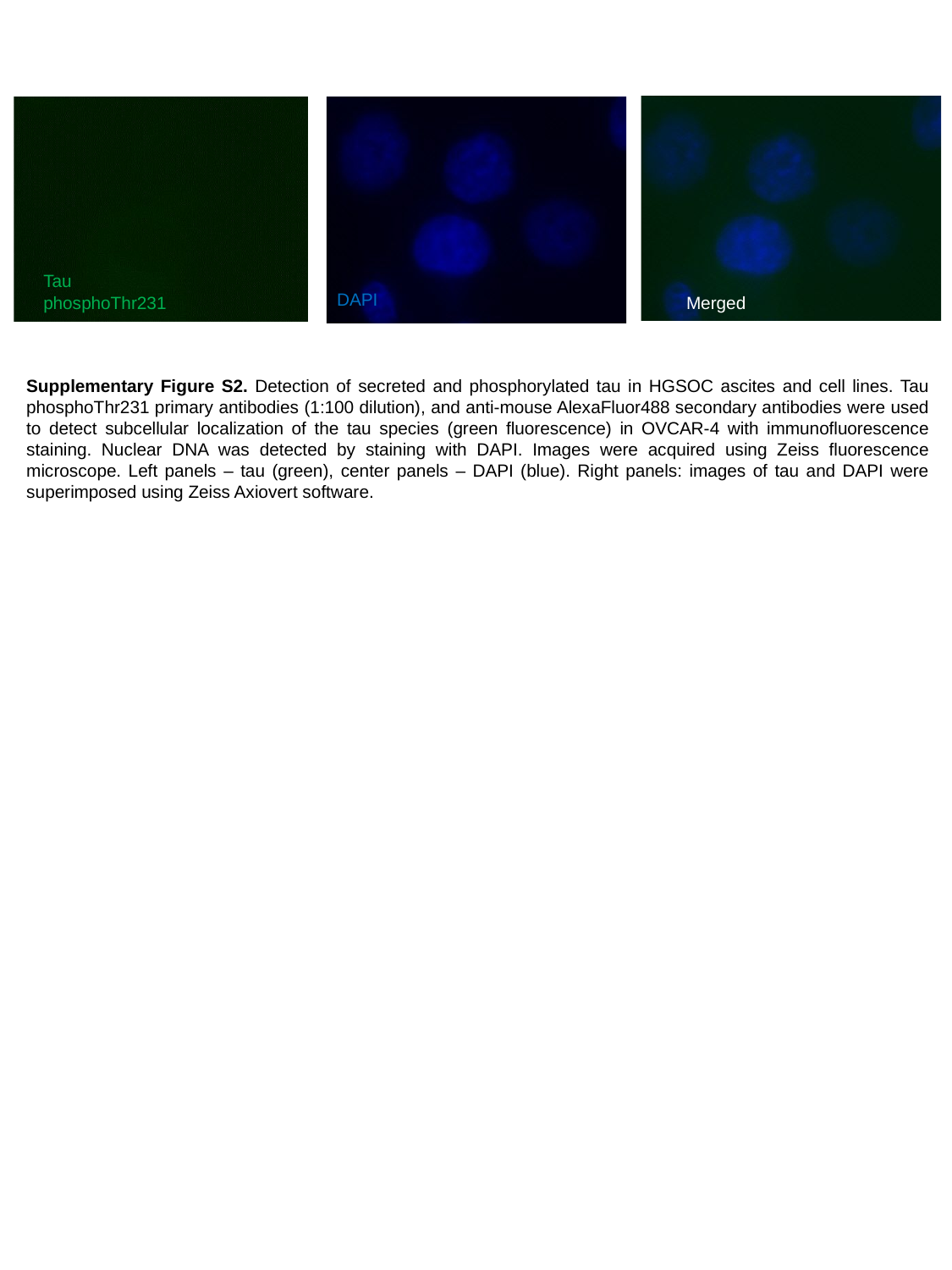

Tau
phosphoThr231
DAPI
Merged
Supplementary Figure S2. Detection of secreted and phosphorylated tau in HGSOC ascites and cell lines. Tau phosphoThr231 primary antibodies (1:100 dilution), and anti-mouse AlexaFluor488 secondary antibodies were used to detect subcellular localization of the tau species (green fluorescence) in OVCAR-4 with immunofluorescence staining. Nuclear DNA was detected by staining with DAPI. Images were acquired using Zeiss fluorescence microscope. Left panels – tau (green), center panels – DAPI (blue). Right panels: images of tau and DAPI were superimposed using Zeiss Axiovert software.
