# Supplementary material for: Targeting Microtubule-Associated Protein Tau in Chemotherapy-Resistant Models of High-Grade Serous Ovarian Carcinoma
Source: Cancers (Basel). 2022 Sep 19;14(18):4535. doi: 10.3390/cancers14184535 (PMC9496900; doi:10.3390/cancers14184535)
Supplement: Supplementary file 1 [file cancers-14-04535-s001.zip › Supplementary Figure S3.pptx]

## Slide 1
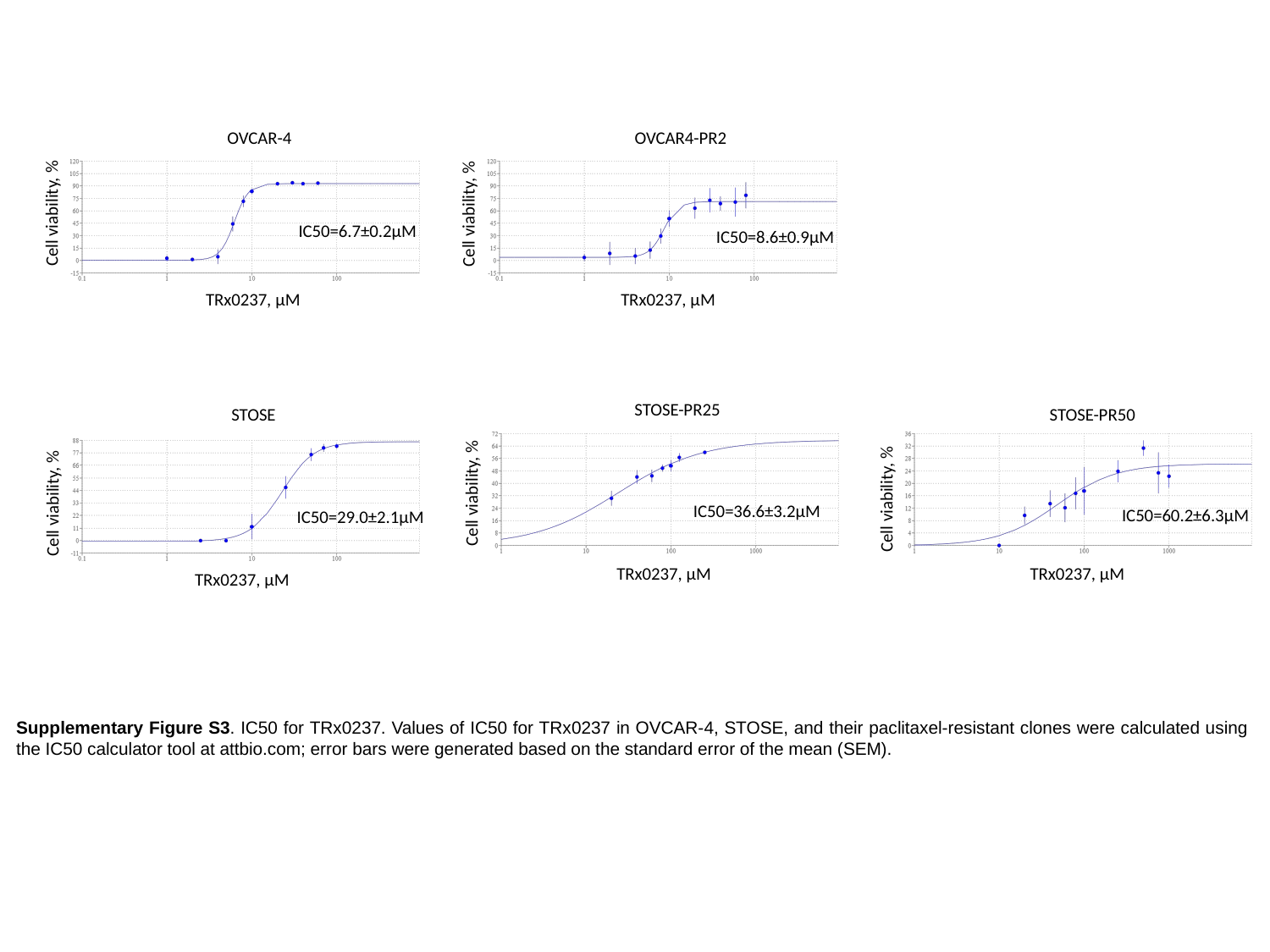

OVCAR-4
Cell viability, %
TRx0237, µM
OVCAR4-PR2
Cell viability, %
TRx0237, µM
IC50=6.7±0.2µM
IC50=8.6±0.9µM
STOSE-PR25
Cell viability, %
TRx0237, µM
STOSE
Cell viability, %
TRx0237, µM
STOSE-PR50
Cell viability, %
TRx0237, µM
IC50=36.6±3.2µM
IC50=60.2±6.3µM
IC50=29.0±2.1µM
Supplementary Figure S3. IC50 for TRx0237. Values of IC50 for TRx0237 in OVCAR-4, STOSE, and their paclitaxel-resistant clones were calculated using the IC50 calculator tool at attbio.com; error bars were generated based on the standard error of the mean (SEM).
