# Supplementary material for: Targeting Microtubule-Associated Protein Tau in Chemotherapy-Resistant Models of High-Grade Serous Ovarian Carcinoma
Source: Cancers (Basel). 2022 Sep 19;14(18):4535. doi: 10.3390/cancers14184535 (PMC9496900; doi:10.3390/cancers14184535)
Supplement: Supplementary file 1 [file cancers-14-04535-s001.zip › Supplementary Figure S4.pptx]

## Slide 1
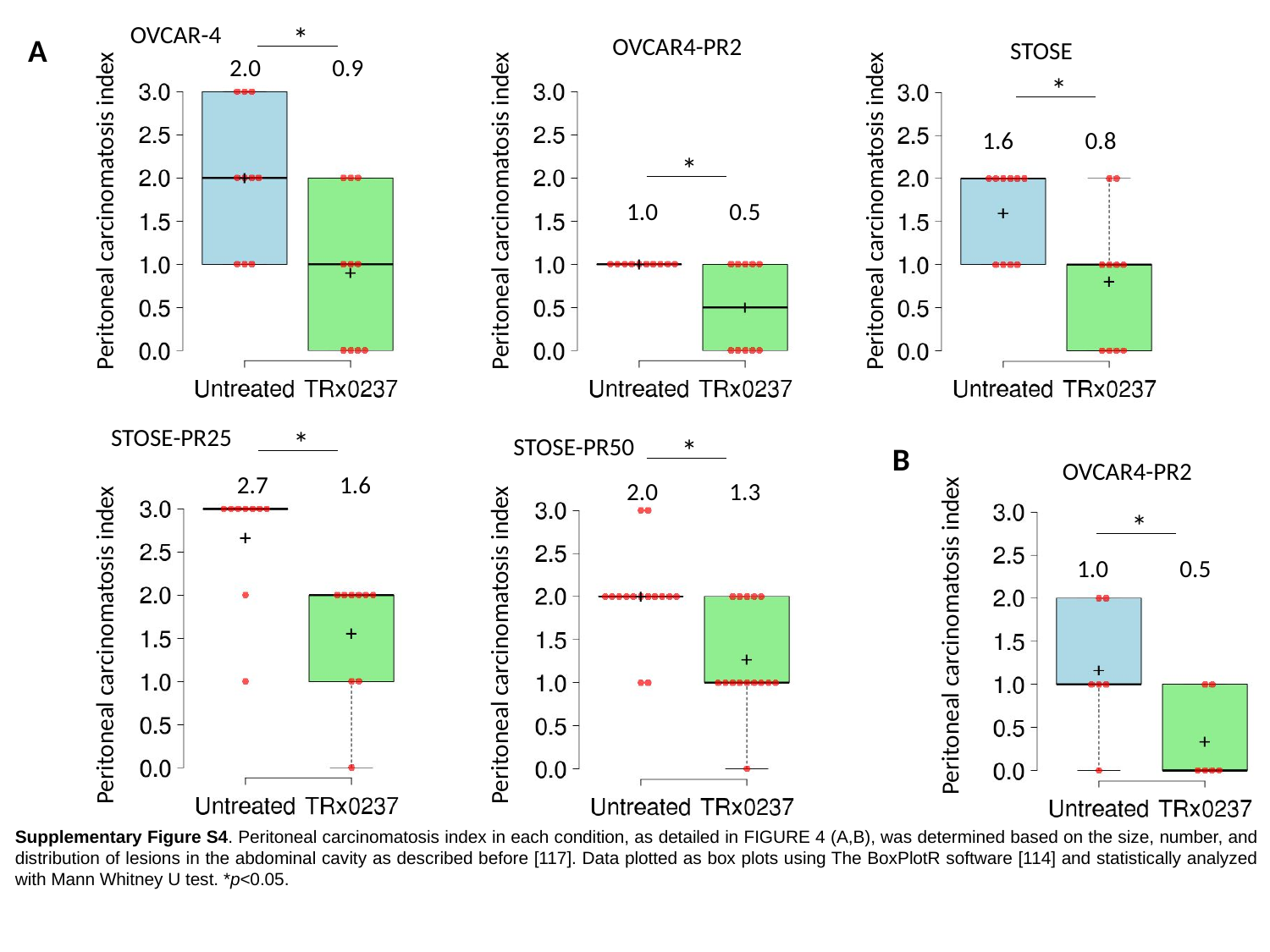

*
OVCAR-4
2.0 0.9
Peritoneal carcinomatosis index
A
OVCAR4-PR2
*
1.0 0.5
Peritoneal carcinomatosis index
STOSE
*
1.6 0.8
Peritoneal carcinomatosis index
STOSE-PR25
*
2.7 1.6
Peritoneal carcinomatosis index
STOSE-PR50
*
2.0 1.3
Peritoneal carcinomatosis index
B
OVCAR4-PR2
*
1.0 0.5
Peritoneal carcinomatosis index
Supplementary Figure S4. Peritoneal carcinomatosis index in each condition, as detailed in FIGURE 4 (A,B), was determined based on the size, number, and distribution of lesions in the abdominal cavity as described before [117]. Data plotted as box plots using The BoxPlotR software [114] and statistically analyzed with Mann Whitney U test. *p<0.05.
