# Supplementary material for: Targeting Microtubule-Associated Protein Tau in Chemotherapy-Resistant Models of High-Grade Serous Ovarian Carcinoma
Source: Cancers (Basel). 2022 Sep 19;14(18):4535. doi: 10.3390/cancers14184535 (PMC9496900; doi:10.3390/cancers14184535)
Supplement: Supplementary file 1 [file cancers-14-04535-s001.zip › Supplementary Figure S5.pptx]

## Slide 1
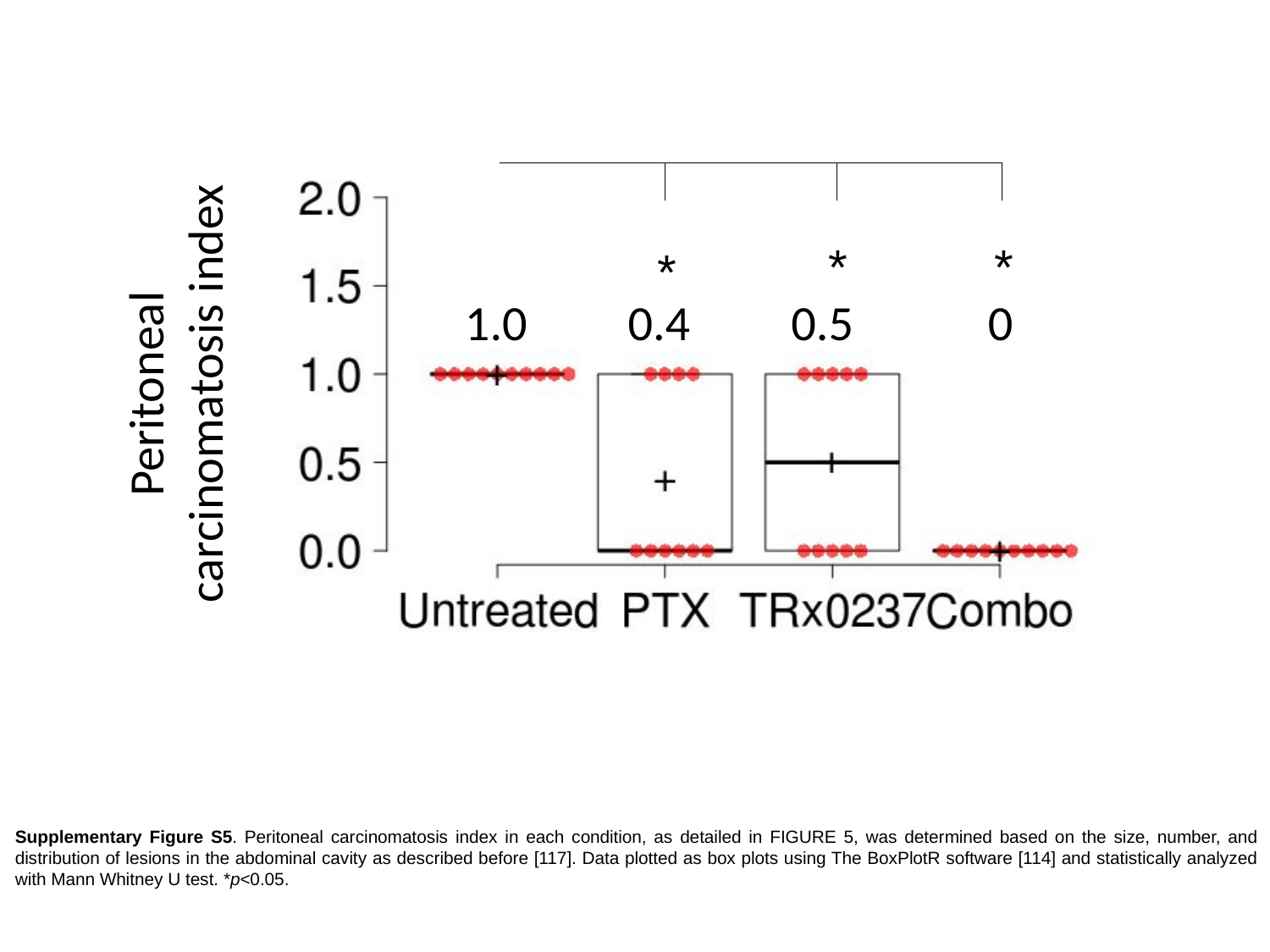

*
*
*
1.0 0.4 0.5 0
Peritoneal carcinomatosis index
Supplementary Figure S5. Peritoneal carcinomatosis index in each condition, as detailed in FIGURE 5, was determined based on the size, number, and distribution of lesions in the abdominal cavity as described before [117]. Data plotted as box plots using The BoxPlotR software [114] and statistically analyzed with Mann Whitney U test. *p<0.05.
