# Supplementary material for: Targeting Microtubule-Associated Protein Tau in Chemotherapy-Resistant Models of High-Grade Serous Ovarian Carcinoma
Source: Cancers (Basel). 2022 Sep 19;14(18):4535. doi: 10.3390/cancers14184535 (PMC9496900; doi:10.3390/cancers14184535)
Supplement: Supplementary file 1 [file cancers-14-04535-s001.zip › Supplementary Table S2.pptx]

## Slide 1
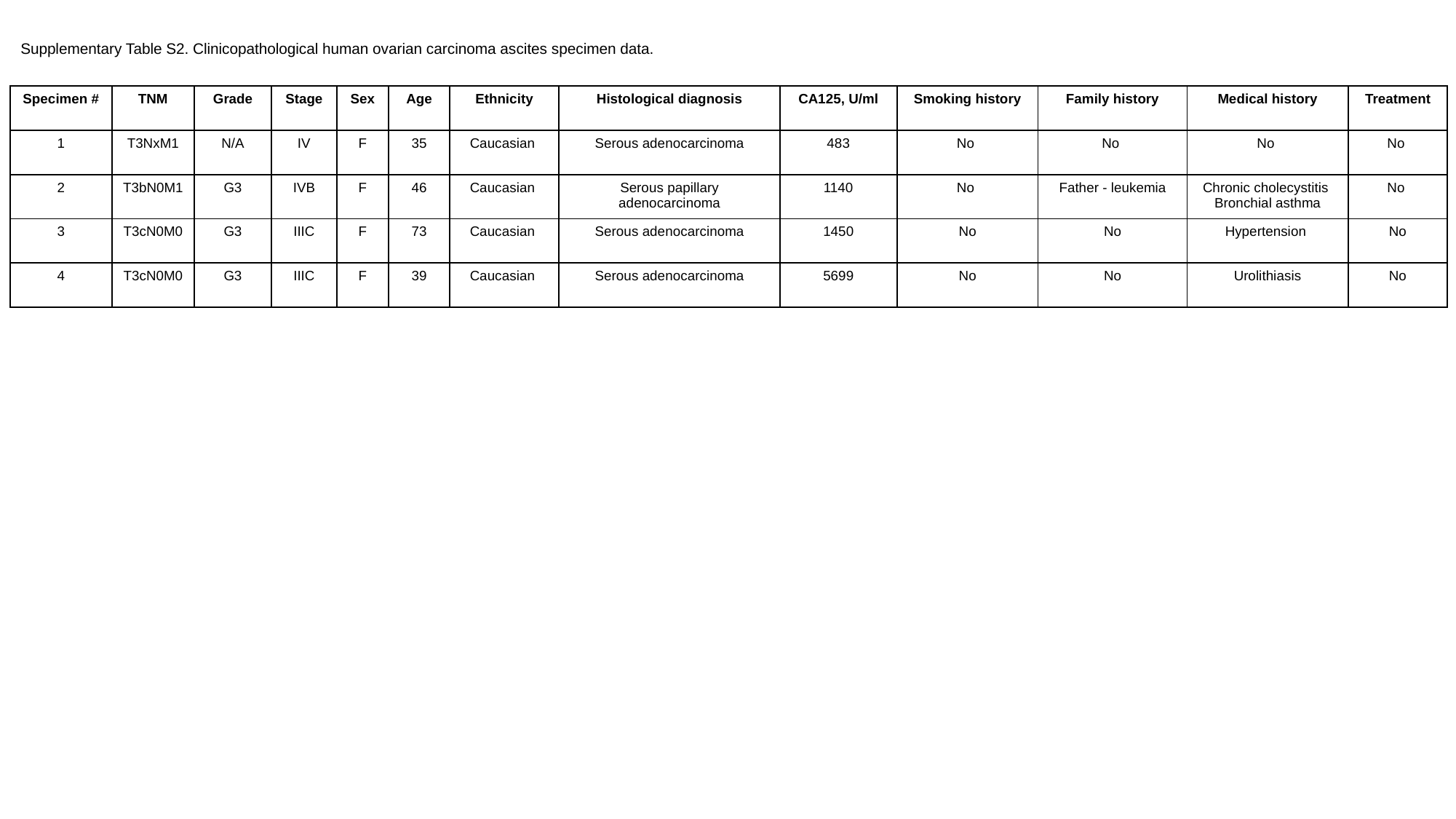

Supplementary Table S2. Clinicopathological human ovarian carcinoma ascites specimen data.
| Specimen # | TNM | Grade | Stage | Sex | Age | Ethnicity | Histological diagnosis | CA125, U/ml | Smoking history | Family history | Medical history | Treatment |
| --- | --- | --- | --- | --- | --- | --- | --- | --- | --- | --- | --- | --- |
| 1 | T3NxM1 | N/A | IV | F | 35 | Caucasian | Serous adenocarcinoma | 483 | No | No | No | No |
| 2 | T3bN0M1 | G3 | IVB | F | 46 | Caucasian | Serous papillary adenocarcinoma | 1140 | No | Father - leukemia | Chronic cholecystitis Bronchial asthma | No |
| 3 | T3cN0M0 | G3 | IIIC | F | 73 | Caucasian | Serous adenocarcinoma | 1450 | No | No | Hypertension | No |
| 4 | T3cN0M0 | G3 | IIIC | F | 39 | Caucasian | Serous adenocarcinoma | 5699 | No | No | Urolithiasis | No |
