# Supplementary material for: Targeting Microtubule-Associated Protein Tau in Chemotherapy-Resistant Models of High-Grade Serous Ovarian Carcinoma
Source: Cancers (Basel). 2022 Sep 19;14(18):4535. doi: 10.3390/cancers14184535 (PMC9496900; doi:10.3390/cancers14184535)
Supplement: Supplementary file 1 [file cancers-14-04535-s001.zip › Supplementary Table S1.pdf]

SUPPLEMENTARY TABLE S1. IC50 for paclitaxel and carboplatin.

Cell line	IC50 paclitaxel, nM	IC50 carboplatin, μM
OVCAR-4	4.1±0.6	111.0±2.8
STOSE	70.3±5.1	126.7±13.7
OVCAR4-PR2	20.5±1.2	98.0±3.4
STOSE-PR25	216.6±10.0	nd
STOSE-PR25-3	177.7±8.6	nd
STOSE-PR50	265.2±13.6	nd
STOSE-PR50-2	200.3±10.6	nd
STOSE-PR50-3	214.8±16.4	nd

nd – not determined

The data are an average of 4 independent experiments conducted with 8 replicas ± standard deviation.
